# Design Optimization of Printed Multi-Layered Electroactive Actuators Used for Steerable Guidewire in Micro-Invasive Surgery

**DOI:** 10.3390/ma17092135

**Published:** 2024-05-02

**Authors:** Simon Toinet, Mohammed Benwadih, Helga Szambolics, Christine Revenant, David Alincant, Marine Bordet, Jean-Fabien Capsal, Nellie Della-Schiava, Minh-Quyen Le, Pierre-Jean Cottinet

**Affiliations:** 1 University Grenoble Alpes, CEA, LITEN DTNM, 38000 Grenoble, France; simon.toinet@cea.fr (S.T.); mohammed.benwadih@cea.fr (M.B.); helga.szambolics@cea.fr (H.S.); christine.revenant@cea.fr (C.R.); david.alincant@cea.fr (D.A.); 2 Department of Vascular and Endovascular Surgery, Hospices Civils de Lyon, 69500 Bron, France; marine.bordet@chu-lyon.fr (M.B.); nellie.della-schiava@chu-lyon.fr (N.D.-S.); 3 LGEF Laboratory, INSA Lyon, UR682, 69621 Villeurbanne, France; jean-fabien.capsal@insa-lyon.fr

**Keywords:** steerable smart guidewire, flexible actuator, multi-layer beam, electroactive polymer, screen-printing, design guideline, medical application

## Abstract

To treat cardiovascular diseases (i.e., a major cause of mortality after cancers), endovascular-technique-based guidewire has been employed for intra-arterial navigation. To date, most commercially available guidewires (e.g., Terumo, Abbott, Cordis, etc.) are non-steerable, which is poorly suited to the human arterial system with numerous bifurcations and angulations. To reach a target artery, surgeons frequently opt for several tools (guidewires with different size integrated into angulated catheters) that might provoke arterial complications such as perforation or dissection. Steerable guidewires would, therefore, be of high interest to reduce surgical morbidity and mortality for patients as well as to simplify procedure for surgeons, thereby saving time and health costs. Regarding these reasons, our research involves the development of a smart steerable guidewire using electroactive polymer (EAP) capable of bending when subjected to an input voltage. The actuation performance of the developed device is assessed through the curvature behavior (i.e., the displacement and the angle of the bending) of a cantilever beam structure, consisting of single- or multi-stack EAP printed on a substrate. Compared to the single-stack architecture, the multi-stack gives rise to a significant increase in curvature, even when subjected to a moderate control voltage. As suggested by the design framework, the intrinsic physical properties (dielectric, electrical, and mechanical) of the EAP layer, together with the nature and thickness of all materials (EAP and substrate), do have strong effect on the bending response of the device. The analyses propose a comprehensive guideline to optimize the actuator performance based on an adequate selection of the relevant materials and geometric parameters. An analytical model together with a finite element model (FEM) are investigated to validate the experimental tests. Finally, the design guideline leads to an innovative structure (composed of a 10-stack active layer screen-printed on a thin substrate) capable of generating a large range of bending angle (up to 190°) under an acceptable input level of 550 V, which perfectly matches the standard of medical tools used for cardiovascular surgery.

## 1. Introduction

### 1.1. Guidewires Used for Cardiovascular Surgery

The function of the cardiovascular system is to distribute oxygen and nutrients to the organs through the bloodstream. It connects the various organs of the human body via a circuit of vessels called veins and arteries [1]. To treat some pathologies, surgeons needs to navigate within these vessels to reach the target organ or tissue [2,3]. This type of intervention is part of MIS (Micro-Invasive Surgery), which has been in full operation for the last twenty years. Based on image-guided systems, MIS reduces the morbidity and mortality of surgical procedures, the operating time, the risk of infection, and the patient’s recovery time [4,5]. The cardiovascular system has angulations of up to 120°. To navigate and catheterize, surgeons frequently use guidewire and various angled non-steerable catheters. There exist a few steerable catheters with a push-and-pull system, but they are too cumbersome to handle and manipulate in several vessels [6,7,8]. A steerable guidewire could eliminate the need for angled catheters, reduce the number of manipulations, thereby reducing the risk of vessel lesions and the global costs. Such a guidewire could be used thousands of times a day all over the world by cardiologists, vascular surgeons, neuroradiologists, etc. Moreover, with the development of robot-assisted surgery, alternative methods for actively controlling the curvature of a catheter or guidewire have emerged.

To actively control a guidewire, several technologies been extensively explored, including hydraulic [9,10], magnetic [11,12,13], concentric tube [14], smart material alloys (SMA) [15,16], electroactive polymers (EAP) [17], piezoelectric ceramics [18], and so on [19]. Among all kinds of actuators, piezoelectric ceramics, despite their excellent electromechanical coupling, are not suitable because of their high stiffness and low maximum displacement [20,21,22]. Composites combining piezoelectric particles and polymer matrix could be an alternative. Nonetheless, further complex procedures are needed to obtain adequate mixtures, which depend on several factors like the concentration, the shape, the size, and the nature of particles. The mechanical and dielectric properties of the polymer matrix, as well as its dispersion with the particles also impact the composite characteristics. In general, the composites exhibit weaker piezoelectric activity with respect to the EAP, as the electric field is more concentrated in the matrix rather than in the ceramic particles [23]. The EAP, with its excellent flexibility, high mechanical strain, and energy density, is, thus, investigated in this study for the development of a smart steerable catheter. Two types of commercially available EAPs are selected including PVDF-TrFE copolymer (known for its ferroelectric properties [24,25,26]), and PVDF-TrFE-CTFE terpolymer (known to be a relaxor ferroelectrics [27,28]). To the best of our knowledges, research has been already carried out on those PVDF-based steerable devices for medical issue [29]. However, none of them have shown the influence of the geometric and material parameters on the actuation ability, which, to some extent, is of enormous significance to optimize the device performance. Keeping in mind the needs of surgeons as well as the medical standards for the use of electrical devices in the human body, a set of design rules (a so-called framework) for a steerable guidewire actuator is thoroughly investigated.

### 1.2. Framework Used for Printed Electronics (PEs)

As printed electronics (PEs) continue to advance, there is a need for design methods to direct innovations for achieving further multifunctional structures using electroactive polymer (EAP) [30,31,32,33,34,35,36,37]. Unfortunately, recent research suggests that the PE technologies remain underutilized in a large industrial scale [38,39,40] because of unpracticable and complex processes. Therefore, design for printed electronics (DfPE) is necessary to provide a framework that facilitates decision making as well as practical integration [41,42,43,44]. DfPE is a multifaceted field of study in which diverse topics such as mechanical and electrical engineering, materials science, optimization, and validation are all considered for enhancing PEs via holistic approaches [45,46,47,48]. Advances in DfPE are, thus, necessary to keep up with the exponential increase in PE applications, especially in medical-field-based printing of additive manufacturing (AM) [49,50,51]. Here, DfPE research is surveyed based on the development of a multi-layer actuator for a smart guidewire, with a particular focus on the design strategy ultimately related to medical use.

**Figure 1:**
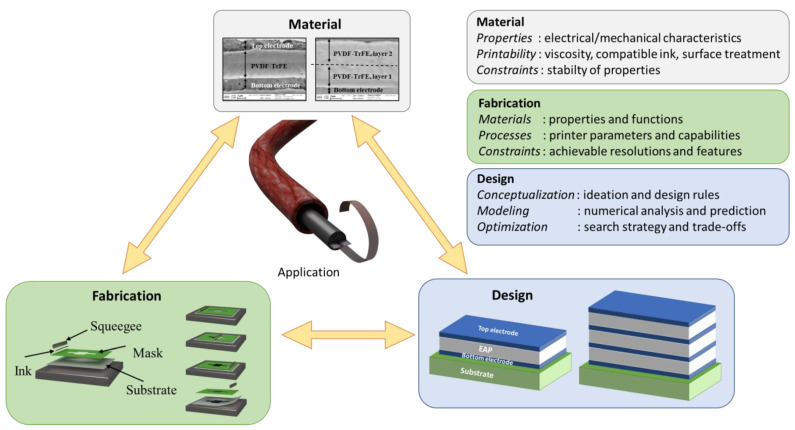
Framework of printed electronics used for the development of a smart actuator-based EAP.

**Figure 2:**
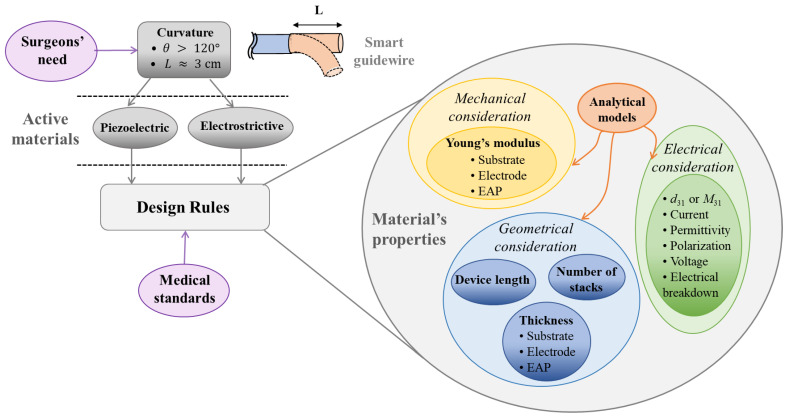
Diagram of different actors and parameters related to the steerable guidewire.

A key aspect of DfPE performed in this study involves the establishment of a framework to implement relevant surgical tools that depends on three main stages consisting of material, fabrication, and design, as illustrated in Figure 1. Regarding the target application, it is potentially best to start with the identification of high-pertinence materials (e.g., piezoelectric and/or electrostrictive polymers) that are adaptable to 3D printing AM, biocompatible and sustainable in the surgical field, and meet the medical standard of MIS (Micro-Invasive Surgery) thanks to their adequate properties. Those properties (including mechanical, electrical, and geometrical considerations, as detailed in Figure 2) are then figured out and characterized using design rules together with experimental tests supported by analytical and numerical solutions. Among possible design configurations, the most appropriate one is chosen for the development of a final prototype, which must be feasible for the fabrication process as well as fulfill the medical specifications.

**Figure 3:**
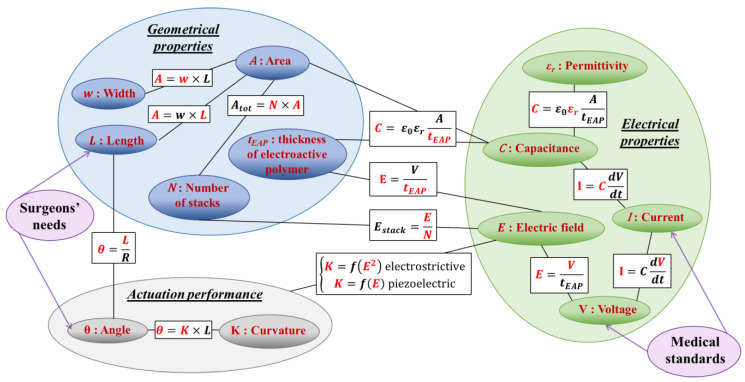
Inter-dependence diagram of electrical and geometrical parameters that have an impact on the actuator performance.

Figure 3 highlights the intra-dependence (relations of parameters inside each category) and inter-dependence (relations of parameters between categories) of the geometrical and electrical properties, which, in turn, strongly affect the device’s flexibility and its actuation performance. The proposed guideline, i.e., based on the adjustment of those parameters, gives an efficient way to achieve the desired goal. For instance, to improve the curvature (an indicator of the bending ability) of the actuator, the input electric field could be increased, which is, however, indirectly limited by the medical standards (60601-1). An alternative involves an increase in the number of stacks that might somehow alter the flexibility of the device. To sum up, the intra- and inter-dependence of all parameters implies a complex dimensioning method, with which compromises among criteria should be considered. Given the large number of parameters that influence the curvature (Figure 2) and their dependences (Figure 3), this paper aims to demonstrate that the nature and geometry of the materials (including active layer and substrate), as well as the manufacturing process, do have strong impacts on the curvature of a steerable guidewire.

## 2. Materials and Methods

### 2.1. Material Selection

In this study, two different substrates were used consisting of PEN (polyethylene-naphthalene) and PI (polyimide). PEN was provided by DUPONT (Teonex DuPont Teijin Films Teonex Q65FA) in thickness of 125 µm and 50 µm. PI was purchased from UBE Japan with several thickness of 125 µm, 50 µm, and 25 µm. PI has been used in medical fields thanks to its highly biomedical grade [52,53,54].

Two dielectric electroactive polymers (EAPs) are investigated. First, poly(vinylidene fluoride-trifluoroethylene) copolymer (80–20%) P(VDF-TrFE), with FC20P grade, is described as a ferroelectric polymer when crystallized in β-phase [55]. Second, the poly(vinylidene fluoride-trifluoroethylene-chlorotrifluoroethylene) terpolymer (61–31–8%) PVDF-TrFE-CTFE, with RTTS grade, is depicted as a relaxor ferroelectric with high electrostrictive properties [56]. Both the copolymer and terpolymer were synthetized by Arkema Piezotech (Lyon, France).

To create the electrical contacts, electrodes made of PEDOT:PSS (CLEVIOS SV4 STAB) conductor (i.e., provided by Heraeus) are coated on both sides of the EAP surface. Such a material was chosen thanks to its high electrical conductivity as well as large mechanical elasticity. These features allow PEDOT:PSS to create reliable electrical contacts without cracking, despite significant deformation could be driven by the actuator [57,58].

### 2.2. Fabrication Process

**Figure 4:**
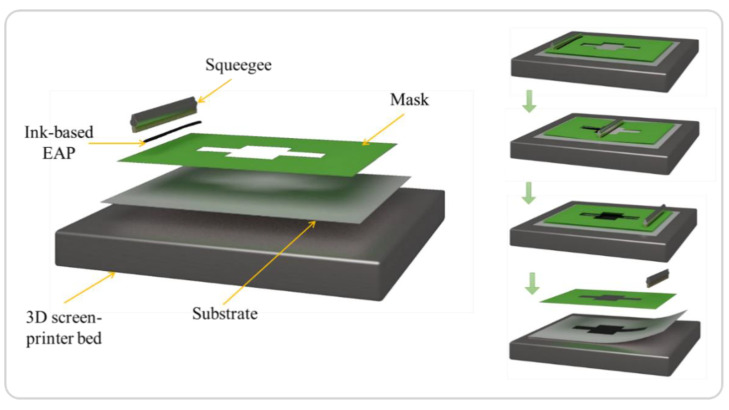
Different steps of screen-printing for additive manufacturing (AM) using EAP ink. Each green arrow corresponds to one step of the deposition of each layer (substrate, EAP, electrode).

The multi-layer device is composed of a flexible/passive substrate, on which an EAP layer between two electrodes is screen-printed via serigraphy. Screen-printing is an inexpensive and simple method capable of depositing thin layers (a few microns) into a stacked architecture. The mask (also known as a screen) is first placed on the substrate and then the ink is loaded onto the mask. A squeegee is moved across the mask to fill the apertures with ink along a line of contact. The ink is then pulled out of the mask apertures to be deposited on the substrate (Figure 4). One layer is printed at a time, so several masks can be used to produce a multi-layered design. The use of three masks (namely, A, B, and C, whose characteristics presented in Table 1), purchased from Koenen, allows us to vary the thickness of the printed layer. It is also possible to carry out several passes of the squeegee to obtain the desired thickness.

**Figure 5:**
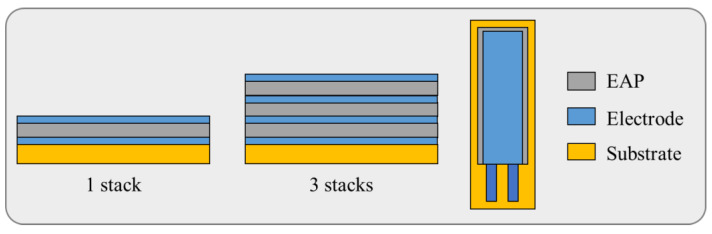
Cross section and plane views of 1-stack and 3-stack devices.

To crystallize the electroactive polymer, thermal annealing was employed under different conditions of temperature (ambient, 70 °C, or 150 °C) and pressure (ambient or quasi-vacuum of ~100 Pa). When stacking the electroactive layers (to obtain a thicker one), each of them needs to be crystallized before being stacked by another one. Therefore, some of the layers are annealed several times. The basic device (i.e., 1 stack), shown in Figure 5, is composed of a substrate on which was deposited a electroactive polymer sandwiched between two electrodes. This architecture can be stacked together to create a multi-layer actuator (e.g., 3 stacks) where the layers are electrically connected in parallel. Such a design results in a lower input voltage application, which is of great interest for medical applications. The electrodes are screen-printed with a thin thickness of 1 µm. To avoid short-circuits, the area of electrodes is designed somewhat smaller than that of the electroactive layer.

**Figure 6:**
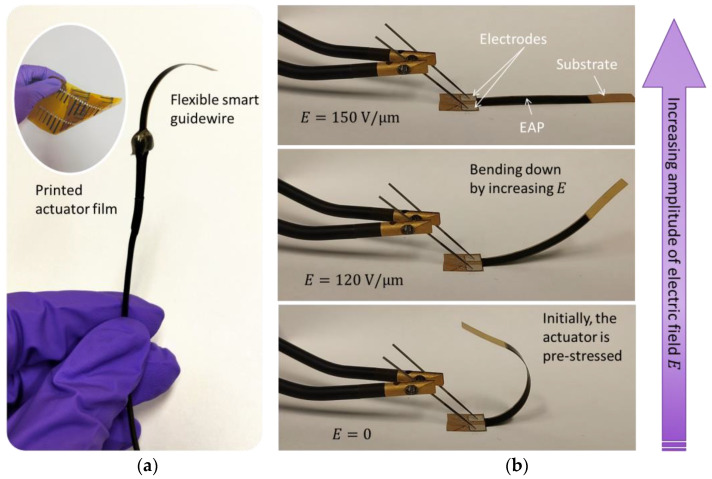
Photography of real actuator films printed using screen-printing: (**a**) flexible smart guidewire; (**b**) actuation driven under different level of the input electric field.

As shown in Figure 5, the actuator is rectangular and slender like a beam structure, whose geometric parameters would substantially affect the deflection of the device. A deep analysis of some of those parameters will be investigated in Section 4, consisting of the nature and thickness of the passive substrate as well as the active electroactive layer, the number of the stacks, and the device slenderness (i.e., defined as a ratio between length and width). Figure 6 illustrates a real prototype developed through the screen-printing AM.

### 2.3. Experimental Characterization Methods

#### 2.3.1. Imaging Microscopy Using AFM and SEM

To visualize the morphology of different printed layers consisting of substrate, EAP, and electrodes, image acquisition via Scanning Electron Microscopy (SEM) was performed. Observation tests were conducted on the cross section of the samples using SEM LEO (ZEISS) equipment (ZEISS, Rueil Malmaison, France). These observations clearly give better comprehension regarding the interaction between the layers’ interface, allowing us to validate the print quality, which is essential for the developed design.

To evaluate the finish and the roughness of the surface of the electroactive layer (i.e., made of copolymer crystalized by vacuum annealing), microscopic images were generated via atomic force microscopy (AFM) using the three masks A, B, and C (cf., Table 1). The experimental setup is based on a commercial AFM (Veeco Dimension 3000, Houghton, Michigan, USA) that generates numerical images acquired via tapping-mode AFM.

#### 2.3.2. Structural Characterization Using XRD

This study was followed by morphological analysis of the sample via X-ray diffraction (XRD) manipulated via a D8 diffractometer (Bruker, MA, USA). The XRD setup was taken in continuous mode, over a range of 10°–90° angles, with carbon-filtered CuKα (1.5406 Å) source.

#### 2.3.3. Electrical Characterization

Broadband dielectric spectroscopy of the samples was performed using an Agilent E4980A Impedance Meter (Keysight, California, USA) at ambient temperature. The dielectric spectra were acquired using an AC electric field with an amplitude of 1±0.1 Vrms and a frequency range of 20 Hz to 2 MHz. The dielectric permittivity was calculated from the measured capacitance using the formular of a planar capacitor whose dimension was known. Based on the sample architecture in which the active element is sandwiched between the top and bottom electrodes, it is clear that the measurement does not depend on the thick substrate.

Polarization hysteresis cycles (i.e., charge density versus electric field) were obtained using the Precision Multiferroic II Ferroelectric Tester (Radiant Technologies Inc., Albuquerque, USA). To evaluate the leakage currents due to the conduction effect [59], the samples were conducted under a DC voltage with a magnitude of 20 V/µm.

#### 2.3.4. Mechanical Characterization

**Figure 7:**
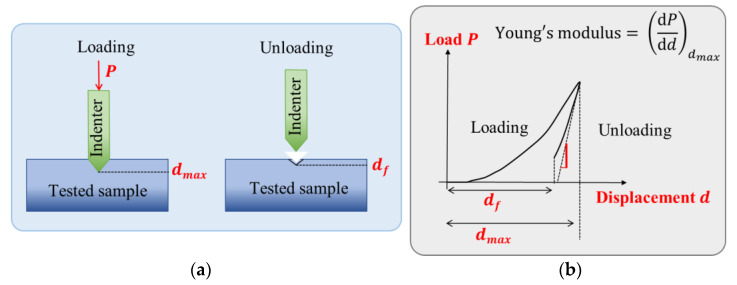
Principal of mechanical characterization: (**a**) schematic diagram of a nanoindentation; (**b**) typical curve obtained for this type of measurement.

Young’s modulus of the different materials was determined via nanoindentation, the most applied means of testing the mechanical properties of small volumes of materials, e.g., thin films. As illustrated in Figure 7a, the technique consists in pressing a diamond cone indenter of known geometry and harness into a tested sample with an applied load (P), which is increased as the tip penetrates further into the sample. The position of the indenter tip is monitored and recorded continuously. The load data P, as a function of the penetration depth (denoted d), can be used to determine the Young’s modulus of the sample, which corresponds to the slope of the curve $\frac{dP}{dd}$ (see Figure 7b).

#### 2.3.5. Tip Deflection Based on Bending Measurements

**Figure 8:**
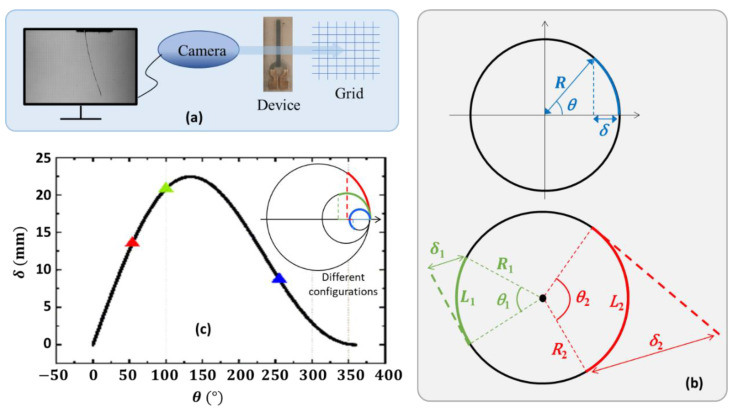
(**a**) Schematic diagram for measuring a sample (appeared as side view on the monitor) with a displacement superior to 4 mm. (**b**) Geometric parameters describing the curvature (**top**). Diagram of two devices with the same radius of curvature but not the same length (**bottom**). (**c**) Tip displacement as a function of angle and the inset on the top right-hand side illustrates different deflection configurations corresponding to those indicated on the curve (triangle marks in red, green, and blue colors).

The displacement (denoted) of the device tip, for values below 4 mm, was measured using a confocal laser CCS Optima + (STIL SAS, Aix-en-Provence, France). Otherwise (δ>4 mm), we opted for a grid system (graduated every 0.5 mm) coupled to a camera S- (AOS Technologies AG, Dättwil, Switzerland) that was piloted by a computer. As shown in Figure 8a, the beam was placed between the camera and the grid. Its bending can be quantified using various factors (see Figure 8b-top) such as bending angle (*θ*), curvature (*K*), radius of curvature (*R* where R=1/K), and tip deflection (or tip displacement, *δ*). Among them, the most often used are the bending angle and the tip deflection, which, somehow, depend on the length of the device (noted *L*, with L=Rθ). It is, therefore, better to use *K* or *R* if possible (e.g., with a constant curvature or a circular-arc device). The relationship between the tip deflection, the angle of curvature and the length is given by

(1) $$\delta =R\left(1-\cos\theta \right)=\frac{L\left(1-\cos\theta \right)}{\theta }.$$

Figure 8b (bottom) highlights that, even with the same value of the curvature (or radius, $R_{1}=R_{2}$), the two parameters *δ* and *θ* may be different (e.g., $\delta_{1}<\delta_{2}$, $\theta_{1}<\theta_{2}$ as $L_{1}<L_{2}$). Whatever the curvature (different from 0), it is always possible to obtain a wide angle θ, if the length L of the device is sufficient:

(2) L=2πR⇒θ=360°.

To a certain extent, *δ* and *θ* are revealed to not fully characterize the bending performance of the actuator device. Particularly, for the intended surgical application where passages are usually narrow, using the curvature or its radius seems to be preferable. From the practical point of view, however, it is not always feasible since some actuator devices, under voltage application, exhibit an elliptical shape instead of circular arc. As the radius R is not constant in the ellipse, the tip displacement δ is, thus, chosen in this study to feature the actuation ability of the device. Figure 8c shows the evolution of *δ* as a function of *θ*, according to the expression of Equation (1), for a constant length of 31 mm. The inset on the top right-hand side illustrates different deflection configurations corresponding to those indicated on the curve (triangle marks in red, green, and blue colors). Interestingly, the tip displacement reaches a maximum value at an angle of 133° instead of 180° as should be expected in theory. Indeed, increasing δ leads to an increase in the angle as well, for a given constant length of the device.

## 3. Results and Discussion

### 3.1. Calculation

#### 3.1.1. Analytical Model

**Figure 9:**
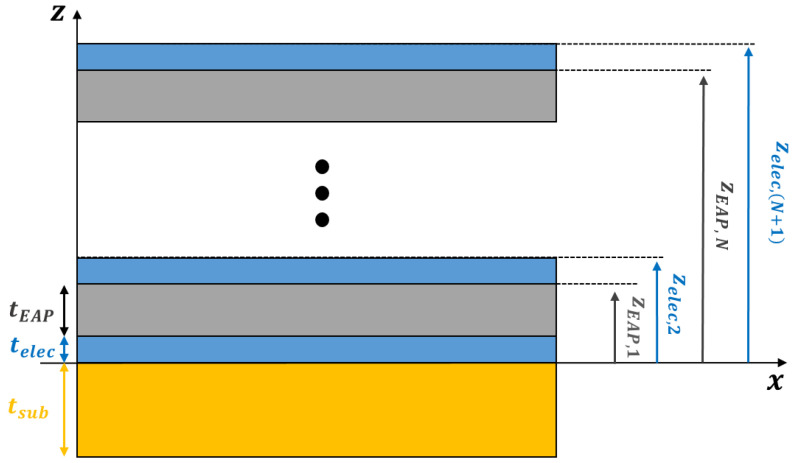
Schematic design of an N-layer actuator comprising electrode–EAP-like architecture. *t_sub_*, *t_elec_*, and *t_EAP_* denote the thickness of the substrate, the (N + 1) electrodes, and the N electroactive layers, respectively. The black dots represent the other layers that are not presented in the image.

To thoroughly understand the bending characteristics of the EAP device, an analytic model was developed in this study. Several electromechanical models have been developed in the literature to calculate the deflection of a multi-layered beam under an applied electric field [60,61,62,63]. Most of them, nonetheless, did not consider the influence of electrodes in the geometry and calculation model [64]. In fact, when the thickness of the electrodes is thin enough (a few tens of nm, when using the sputtering method) compared to that of the active layer (i.e., at the order of micrometric), it can be assumed that their impact on the electromechnical response of the device is minimized. In additive manufacturing (AM) where the electrodes are printed in a comparable thickness to the active layer, the device becomes stiffer as its mechanical property might be changed by the electrodes. In other words, the electrodes create a mechanical constraint on the active layer, reducing its motion when subjected to a given input voltage. Concretely, in our multi-layered specimen (see Figure 9), the electrodes, representing ~10–15% of the total thickness of the sample, should be taken into consideration in the analytical model. The electroactive layers induce compound bending (*S*) that is determined as the superposition of pure traction/compression (*S*_0_) and pure bending (*Kz*) [29,64]:

(3) $$S=Kz+S_{0}.$$

The above model represents compound bending, where *z* and *K* denote the position and the curvature; the *Kz* product describes simple bending [21]. In general, the theoretical model of simple bending is based on the following assumptions [65]:

- Each layer of the beam can be piezoelectric/electrostrictive or purely elastic;
- The device (plate) thickness is negligible with respect to the curvature radius;
- The cross section of the layers is constant along the length of the plate;
- The whole system is in a static equilibrium, in which the stress distribution within the cross section is supposed to be constant, whatever the bending deflection;
- The xz-plane is the plane of symmetry (see Figure 9).

For compound bending, in the case of multi-layered architecture, further hypotheses are made as following:

- The device has a longitudinal (along x-axis) strain and a flexural strain;
- The multi-layer bends in a circular arc [64];
- The neutral axis is the boundary between the substrate and the first electrode [29].

Under those assumptions, the stress of different layers can be written as:

(4) $$\left\{\begin{array}{c} T_{sub}=Y_{sub}\left(Kz+S_{0}\right)\\ T_{elec,k}=Y_{elec}\left(Kz+S_{0}\right)\\ T_{EAP,k}=Y_{EAP}\left(Kz+S_{0}-S_{d}\right) \end{array}\right.$$

where *Y_i_* is the Young’s modulus [Pa]; *K* is the curvature [m^−1^]; *S*_0_ is the longitudinal strain (pure traction/compression), *S*_d_ is the electroactive strain (generated by piezoelectricity or electrostriction); *z_a,k_* denotes the position along the z-axis; and *k* is the number of layers.

For an electrostrictive PVDF-TrFE-CTFE material (without temperature change), the transversal strain and the electric displacement are given by [66]:

(5) $$\left\{\begin{array}{c} S_{31}=M_{31}E^{2}+sT_{31}\\ D_{31}=\varepsilon_{0}\varepsilon_{r}E+2M_{31}T_{31} \end{array}\right.$$

where *M*_31_ is the electrostrictive coefficient; $T_{31}$, $S_{31}$, and $D_{31},$ respectively, denote the stress, the transversal strain, and the electric displacement in direction 1 due to a solicitation in direction 3; *s* is the mechanical compliance (from compliance tensor); *E* denotes the input electric field; *ε*_0_ is the vacuum permittivity; and *ε_r_* is the relative permittivity of the electroactive layer.

In absence of external stress ($T_{31}=0$), the strain is simplified by as

(6) $$S_{31}=M_{31}E^{2}.$$

For a piezoelectric PVDF-TrFE material (without temperature change), the strain and the electric displacement are expressed as a function of the transversal piezoelectric coefficient *d*_31_ [67]:

(7) $$\left\{\begin{array}{c} S_{31}=d_{31}E+sT_{31}\\ D_{31}=\varepsilon_{0}\varepsilon_{r}E+d_{31}T_{31} \end{array}\right.$$

In absence of external stress (*T*_31_), the strain is deduced as

(8) $$S_{31}=d_{31}E.$$

For the complete device, the forces equilibrium is, thus, given by

(9) $$\int_{-t_{sub}}^{0}T_{sub}.dz+\sum_{k=1}^{N+1}\int_{z_{EAP,\left(k-1\right)}}^{z_{elec,k}}T_{elec,k}.dz+\sum_{k=1}^{N}\int_{z_{elec,k}}^{z_{EAP,k}}T_{EAP,k}.dz=0.$$

And, the moment equilibrium is expressed as

(10) $$\int_{-t_{sub}}^{0}T_{sub}.z.dz+\sum_{k=1}^{N+1}\int_{z_{EAP,\left(k-1\right)}}^{z_{elec,k}}T_{elec,k}.z.dz+\sum_{k=1}^{N}\int_{z_{elec,k}}^{z_{EAP,k}}T_{EAP,k}.z.dz=0.$$

The force and moment equilibrium constitutes a system of two equations with two unknowns (*K* and *S*_0_). All calculation details for achieving the literal expressions of those parameters can be found in S2 of Supplementary Materials. These parameters, pertinent to assessing the bending performance of the actuator beam, are discussed in the following. A comparison with the numerical solution via COMSOL Multiphysics (COMSOL France SAS, Grenoble, France) is also investigated, confirming the reliability of the developed analytical model.

**Figure 10:**
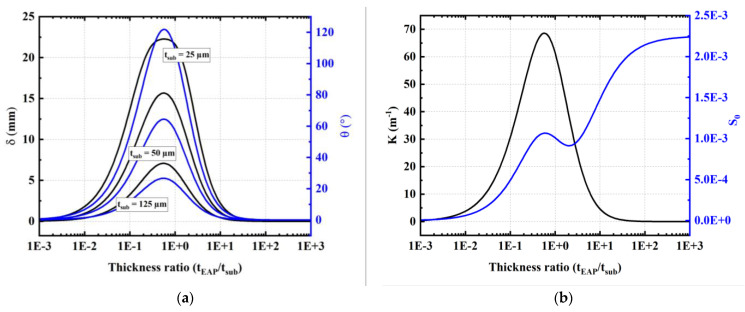
Actuation performance as a function of electroactive/substrate thickness ratio of a 1-stack copolymer/PEN device subjected to a field of 150 V/µm: (**a**) Tip displacement and angle estimated for samples with three different thicknesses of substrate including 25 µm, 50 µm and 125 µm. (**b**) Curvature and elongation determined for a device with 25 µm thick PEN substrate.

Figure 10a depicts the influence of the electroactive/substrate thickness ratio ($t_{EAP}⁄t_{sub}$) on the tip deflection (*δ*) and the bending angle (*θ*). The tested models correspond to one-stack copolymer specimens with variable thickness and 31 mm length, deposited on different PEN substrates with thickness of 25, 50, and 125 µm. All models are powered by an input field of 150 V/µm. Equal-field comparison enables us to stay within the operating window of the EAP, notably below its breakdown field. The results of Figure 10a lead to the below relevant remarks:

- Whatever the thickness of the EAP layer (denoted $t_{EAP}$): the smaller the substrate thickness, the higher the bending angle *θ* (and so is the tip deflection *δ*);
- Both θ and δ have a similar trend, in which their maximum value attains a thickness ratio ($t_{EAP}⁄t_{sub}$) of ~0.6. This finding is somehow coherent to that reported in the literature [68,69];
- Very thin layers give rise to enhanced bending response but could weaken the device structure. Adequate values of both $t_{EAP}$ and $t_{sub}$ should be chosen to achieve the best compromise between the actuation ability and mechanical property of the structure.

As explained above, the total strain is the superposition of the pure normal strain *S*_0_ (inducing elongation) and the simple bending strain S=f(z) that causes the curvature *K*. Figure 10b illustrates both K and *S*_0_ parameters inferred from the theoretical models as a function of the thickness ratio ($t_{EAP}⁄t_{sub}$). The result confirms the optimum of the curvature and the normal strain at a ratio of ~0.6 that also corresponds the maximum value of the bending parameters *θ* and *δ*.

**Figure 11:**
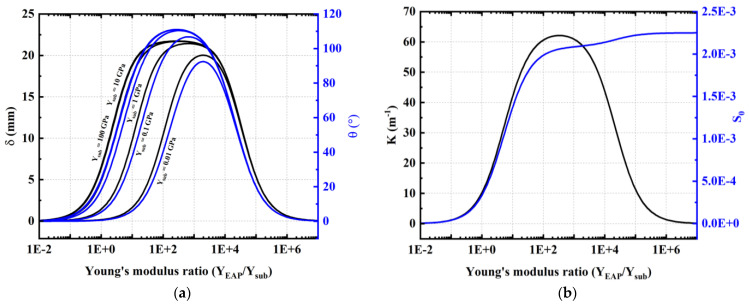
Actuation performance as a function of electroactive/substrate Young’s modulus ratio of a 1-stack, 3 µm thick copolymer device subjected to a 150 V/µm electric field: (**a**) Tip displacement and bending angle of samples with substrate stiffness of 0.01 GPa to 100 GPa; (**b**) Curvature and nominal elongation of a sample with a 1 GPa stiff substrate (this value is chosen as most of commonly used substrates have a stiffness of a few GPa).

Figure 11a highlights the influence of the electroactive/substrate Young’s modulus ratio ($Y_{EAP}⁄Y_{sub}$) on the bending performance. The electroactive layer is 3 µm thick, similar to the one used in Figure 10, while the substrate thickness is fixed at 50 µm and its Young’s modulus ($Y_{sub}$) is set between 0.01 GPa and 100 GPa. As seen, the important parameters are not only the stiffness of the substrate ($Y_{sub})$ but also the ratio $Y_{EAP}⁄Y_{sub}$, upon which the actuation of the system significantly depends. The results of Figure 10 lead to the relevant remarks:

- Whatever the stiffness of the EAP layer (denoted $Y_{EAP}$): the stiffer the substrate, the higher the bending angle *θ* (and so is the tip deflection *δ*). If the substrate Young’s modulus ($Y_{sub}$) exceeds 1 GPa, variation in the angle of curvature and deflection are small (see Figure 10a where the curves with 10 GPa and 100 GPa are superimposed);
- Both *θ* and *δ* have a similar trend, in which their maximum value at the Young’s modulus ratio ($Y_{EAP}⁄Y_{sub}$) is between 300 and 2000 (i.e., corresponding to $Y_{sub}=100$ GPa and 0.01 GPa);
- Too small a value of $Y_{EAP}$ makes the curvature drop off due to low efficiency for the delivery of energy. On the other hand, increasing $Y_{EAP}$ to a significant value provokes a complete elongation of the actuator, whose strain reaches a saturate regime (Figure 10b).

Figure 11a,b confirms the existence of an optimum Young’s modulus ratio that leads to the maximum value for the three bending parameters δ, θ, and K. To achieve such optimum ratios, it is necessary to select the electroactive layer with an important stiffness. As demonstrated in the literature [64,65], adding a significant concentration of inclusions (e.g., piezoelectric ceramics) to the dielectric layer as PVDF could make it stiffer. This, however, would impair the piezoelectric sensitivity as well as drastically change the ink’s viscosity, which somehow makes the printing process challenging. As observed in Figure 11b, below the optimum ratio, increasing the Young’s modulus of the electroactive layer leads to substantially enhanced curvature even if the device elongation (*S*_0_) increases. Above the optimum ratio, the elongation keeps increasing but at a slower rate, while the curvature drastically drops. Indeed, the energy generated by the electroactive layer is mainly used for elongation rather than for bending. From a technological approach, it would be interesting to stack an important number of active layers (comprising an EAP layer sandwiched between two electrodes), so as to boost the tip deflection and the curvature.

**Figure 12:**
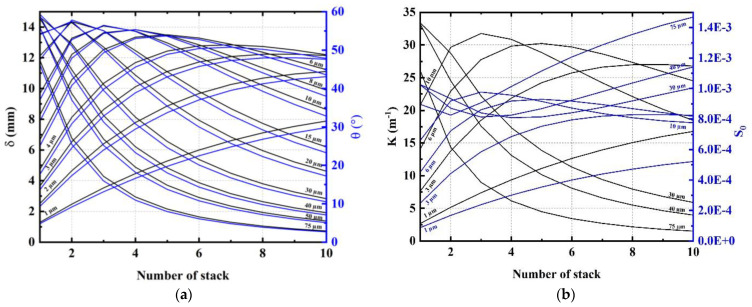
Actuation performance including (**a**) tip displacement; and (**b**) bending angle versus the number of stacks (with thickness from 1 µm to 75 µm, deposited on a 50 µm thick PI substrate) subjected to an electric field of 150 V/µm.

Figure 12a,b depicts the influence of the number and the thickness of the unitary stacks on the bending performance. All samples are subjected to an electric field of 150 V/µm. The same phenomenon appears as in the case of the single-layered device shown in Figure 10: there exists an optimum value for the number of stacks (denoted $N_{stack}$) that depends on its thickness ($t_{stack}$). For thick stacks of 10 µm and 15 µm, the optimum value of $N_{stack}$, respectively, equals 3 and 2 (cf. Figure 12a). For thinner stacks of 3 µm and 4 µm, an optimum occurs at nine stacks and seven stacks, respectively. In any configuration, the total thickness of EAP is almost constant and equals approximately $0.5\times t_{sub}$ to $0.6\times t_{sub}$. In other words, the best bending performance was obtained using the thickness ratio ($t_{EAP}⁄t_{sub}$) of 0.5–0.6, which is coherent with the findings previously revealed in Figure 10. As a result, for a given total EAP thickness, using a large number of electrodes (multi-stack design) instead of two (single-stack design) would be almost equivalent in actuation while lowing the input voltage. However, a drop in the bending performance is observed in Figure 12a, where the maximum displacement decreases as the number of stacks increases. This behavior clearly illustrates one of the design trade-offs: dividing the applied voltage by 10 using a 10-stack device leads to a loss of 20% in the actuation performance. As a matter of fact, introducing more inactive material (electrodes) together with stacking multiple EAP layers would harden the mechanical structure of the device, which, in turn, impedes the device’s displacement. Accordingly, whatever the actuator pattern (either multi- or single-layered, thick or thin stacks/substrate), optimum bending mainly depends on the thickness ratio between the EAP layer and the substrate.

From the practical point of view, however, the geometry choice could also result in important consequences that justify the final decision of the target design. Indeed, a single-layer architecture leads to a simplified and timesaving process but needs a high input voltage to achieve desired bending displacement. Furthermore, a thick sample usually increases the probability of a breakdown field due to higher risk of defects as well as heat-dissipation issues within the thick layer [70,71,72,73]. Regarding the multi-layered structure, the electroactive layers behave as thin capacitors connected in parallel, so they are all powered under the same electric field. Therefore, the multi-layer architecture allows us to expand the range of applications for EAP-based actuators by powering them with a moderate input voltage level (although it adds passive material as electrodes). Such a characteristics clearly confirm the high benefit of using multi-stack design for a steerable guide to navigate the arterial circuit. The analytical model demonstrated that bending of a multi-layer cantilever device is governed by optimization of both geometrical and mechanical factors, while voiding the electrical breakdown effect.

#### 3.1.2. Comparison with Simulation Model

To date, PVDF-based multi-layer cantilever devices modeled using the COMSOL finite element method (FEM) have been widely investigated in the literature [64,65,66,67,68,69,70,71,72,73,74]. To assess the reliability of the simulation, those models were usually compared with experiments but not with an analytical model. In this study, a comparison between the FEM and the new theoretical model described in Section 3.1 is carried out. The simulation model was built based on the “Piezoelectric Devices interface of the Structural Mechanics Module” of COMSOL Multiphysics. The properties of the entire specimen used in FEM are shown in Table 2.

**Figure 13:**
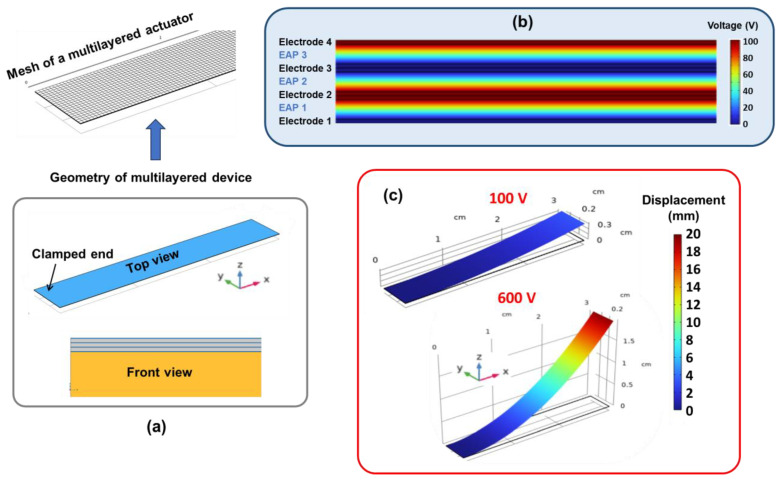
FEM of a 3-stack, actuator-based PVDF-TrFE based on COMSOL software: (**a**) Geometry and meshing. (**b**) Voltage distribution within layers. (**c**) z-displacement at an input voltage of 100 V and 600 V.

Figure 13a shows a top and a side view of a simple geometric design for the multi-layered actuator comprising a PEN substrate and three active layers (PVDF-TrFE copolymer), where each of them is sandwiched between two PEDOT:PSS electrodes. The surface mesh is swept across the thickness of each layer, using at least three meshing elements of 250 µm square size or smaller. The whole cantilever is clamped at one end while the other is free to deform. In the electrostatics node, the “zero charge” condition is applied on all boundaries except the electrodes connecting to the input power supply. To correctly drive the excitation, the voltage is subjected to the four electrodes in an alternating manner. For instance, as illustrated in Figure 13b, electrodes 1 and 3 are set as ground (0 V), whereas electrodes 2 and 4 are set to a positive voltage value. Figure 13c shows the deformation of the system under a voltage of 100 V and 600 V applied to the electrodes. The displacement amplitude is retrieved and then compared to that of experimental/analytical results.

**Figure 14:**
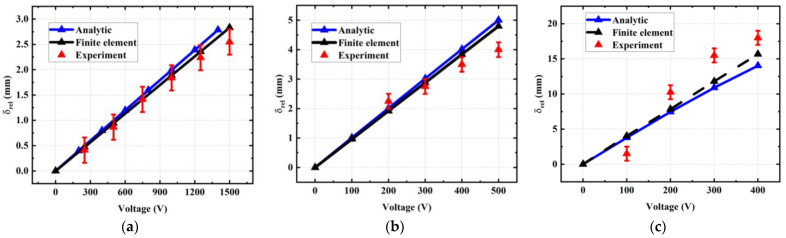
Tip-displacement versus input voltage characteristics obtained through analytical model (blue curves), FEM simulation (black curves), and experiment (red curves). Samples were tested with different variations of stack number and thickness of substrate ($t_{sub}$): (**a**) 1 stack of 9 µm thick deposited on a PEN substrate with $t_{sub}=125\;µm$; (**b**) 10 stacks of 30 µm thick deposited on a PI substrate with $t_{sub}=125\;µm$; (**c**) 10 stacks deposited on a PEN substrate with $t_{sub}=50\;µm$.

Figure 14 shows the tip displacement of the beam as a function of the input voltage obtained using the analytical model (blue curves), FEM (black curves), and experimental measure (red curves). The actuator was designed using either a single- or multi-layered pattern, together with different thickness of the substrate (i.e., 125 µm as in Figure 14a,b and 50 µm as in Figure 14c). Again, the results confirm that, under a given applied voltage, the ten-stack sample allows us to significantly boost the actuation performance as opposed to the one-stack sample. Moreover, thinner substrate leads to an increase in tip displacement, as it is supposed to have less impact on the mechanical property of the EAP layers.

Whatever the design configuration, a linear relationship between the displacement and the voltage occurred, reflecting that the piezoelectric behavior is dominant in the copolymer for the applied electric field less than ~170 V/µm (i.e., corresponding to 1.5 kV applied to a single-stack and 500 V to a 10-stack sample). In addition, the tip displacements computed from both analytical and simulation models are consistent, especially with the thick substrate of 125 µm, regardless of the number of stacks chosen (Figure 14a,b). In the case of thinner substrate, as illustrated in Figure 14c, higher discrepancy between those models was observed, but this is still acceptable with a relative variation less than 10%. Such a discrepancy was probably due to the fact that a 2D geometry was considered in the analytical model while a more complex 3D model was employed in the finite element (FE) simulation. This explains why numerical simulation provides results closer to the real data than analytical theory. It is noteworthy that higher displacement induces higher discrepancy between the models and the experiment. In fact, when bending is driven from the device tip, it takes the shape of an elliptic arc instead of a circular arc. This manifests a different mechanical equilibrium that does not follow the models. To some extent, discrepancies between the real data and the analytical/numerical solutions mainly come from measurement uncertainties, as well as hypotheses and approximation of the geometric/material parameters used in the theory.

### 3.2. Microscopic Image

**Figure 15:**
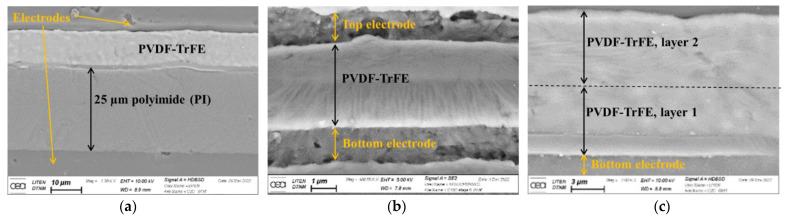
Cross section of the printed sample using SEM with a focus on (**a**) a 25 µm polyimide film; (**b**) copolymer, one pass with mask C (4 µm); (**c**) copolymer, two passes with mask C (8 µm).

Figure 15 shows the cross section of the printed sample using SEM images with a focus on (a) a 25 µm polyimide film, (b) a 4 µm-thick copolymer (one pass with mask C) sandwiched between top and bottom electrodes, and (c) a 8 µm thick copolymer (two passes with mask C). As indicated in Figure 15a, the four layers comprising copolymer, two electrodes, and PI substrate are perfectly stacked together via 3D screen-printing. The substrate exhibits a very smooth surface compared to the other layers. Interestingly, there is a thin blending of PVDF-TrFE and PI formed at the interface between these two layers. No penetration of electrode ink into the copolymer and the substrate allows us to ensure that neither electrical short-circuits nor contact default could occur. Figure 15b confirms that PVDF-based materials consist of a long-chain molecule composed of methylene (CH_2_) and fluorocarbon (CF_2_). The amorphous regions correspond to an irregular arrangement of chain molecules whereas crystallized regions relate to thin lamellar crystal-like structures [67]. The polymorphism of this polymer enables it to crystallize into at least four phases (*α*, *β*, *δ* and *γ*) [75]. Figure 15c is similar to Figure 15b but with a thicker copolymer layer, demonstrating an easy process of screen-printing that allows to us trial different patterns and dimensions of the actuator devices.

**Figure 16:**
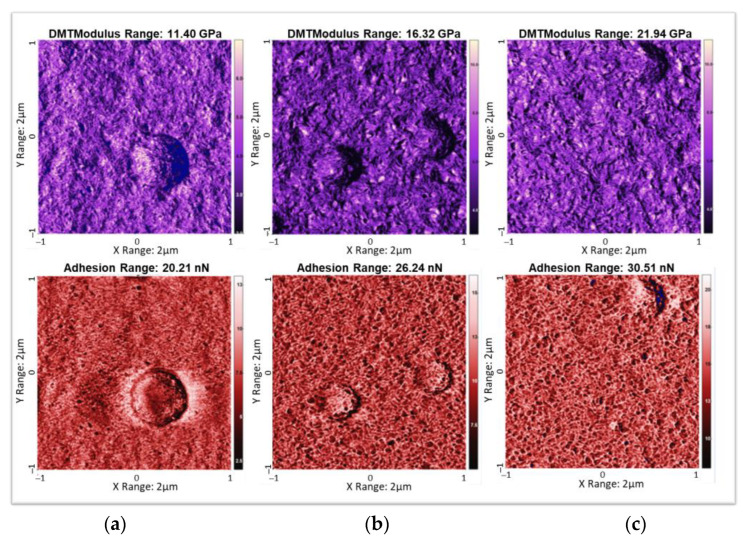
Mapping of DMT modulus (purple AFM images) and adhesion (red AFM images) of PVDF-TrFE crystalized via vacuum annealing and printed through different masks: (**a**) mask A, (**b**) mask B, and (**c**) mask C.

Figure 16 illustrates AFM images of a copolymer device printed with the three masks and crystalized via vacuum annealing. The roughness is found to be equal to 10, 14, and 16 nm, respectively, with the mask A, B, and C. Mask A, with its smallest size of mesh, leads to the best finish of the surface. However, to realize a thick pattern (e.g., electroactive layer), mask C is preferred for achieving fast processing and saving ink (minimizing the number of passes). All masks exhibit less than 20 nm roughness, which is good enough to perform the staking multi-layer design. The absence of holes in the AFM image ensures that there is no defect in the printed actuator. It has been observed that vacuum annealing leads to the best layer state and, therefore, allows for several layers to be stacked.

### 3.3. Structural Analyses

**Figure 17:**
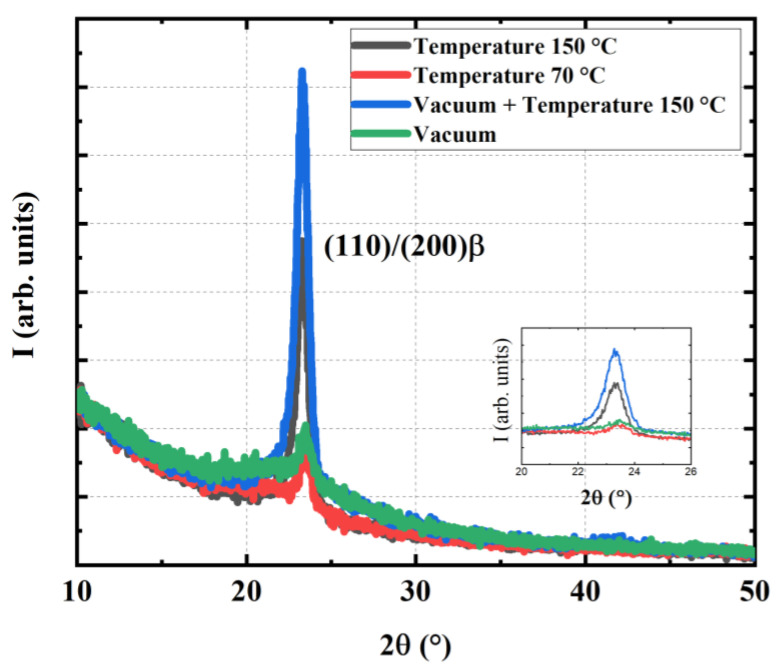
XRD of copolymer films elaborated using different thermal and pressure conditions.

Figure 17 indicates that the annealing method has an influence on the crystallographic structure. It is well-known that the morphology of the terpolymer is more difficult to observe via XRD than that of the copolymer because of its lower crystallinity (between 30% and 40%) [76]. As pointed out by Yuljae et al., polymer annealing favors the appearance of the α-phase, while solvent annealing favors the formation of the β-phase [77]. In general, an irreversible transition from the β-phase to the α-phase occurs between 35 °C and 45 °C. Morphological changes were also observed under electric field conditions, with a transition between 20 and 70 V/µm [78]. From the practical point of view, it is difficult to verify whether this transition is due to a phase change or a rotation of the crystalline domains under the electric field [79]. Vacuum processing leads to homogeneous amorphous layers (no grain boundaries) since the solvent is pumped quickly and freezes [78]. In fact, the electroactive layer is amorphous at the outlet, which is suitable for stacking. Annealing at 70 °C also leads to homogeneous layers but is very time consuming (more than 1 h per layer). Annealing at 150 °C is faster but the polymer is too crystallized to obtain multi-stack structures because of grain boundaries manifesting roughness of the polymer surface. The right compromise is annealing at 150 °C coupled with vacuum, as revealed in Figure 17.

According to our experience, it would avoid thermal annealing under ambient temperature with stacking multi-layers. On a horizontal electroactive surface, the local temperature variation causes changes in interfacial tension, making the motion of the fluid (as polymer is in liquid state) at the interface migrates to the edges. As a result, the electroactive layer is thicker at the edges than in the middle [80,81]. Such a phenomenon, called the Marangoni effect, is an obstacle for the multi-stack design. To some extent, the vacuum allows for a homogeneous layer without grain boundaries. As soon as the annealing temperature is higher than 100 °C (especially 150 °C) and the vacuum is not applied, large grains appear, making multi-stack impossible because of dramatical roughness. The use of vacuum and high temperature results in homogeneous, crystalline layers with little roughness (cf., Figure 16), confirming the reproducibility and reliability of the process.

Based on the data obtained from the X-ray diffraction (XRD), a pertinent parameter relating to the crystallization of polymer, i.e., the domain size (d), is estimated using the following expression:

(11) $$d=\frac{k\lambda }{W_{HM}\times \cos\left(\theta \right)}$$

where *k* the Scherrer constant taken to be 0.9, λ is the X-ray wavelength, $W_{HM}$ is the full width at half maximum of the XRD peak, and θ is the Bragg angle (i.e., shown in Figure 17). As shown in Table 3, the largest size (12 nm) is obtained for solvent extraction under vacuum at 150 °C thermal annealing (Table 3). Again, it can be concluded that optimization of the β-phase ferroelectric for the EAP layers could be achieved through this elaboration method.

### 3.4. Electrical Analyses

**Figure 18:**
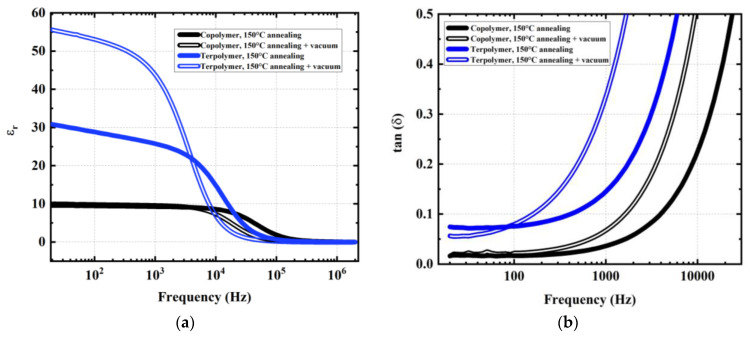
Broadband dielectric measurements of PVDF-TrFE copolymer and PVDF-TrFE-CTFE terpolymer treated using two annealing conditions (ambient pressure and vacuum): (**a**) dielectric permittivity; (**b**) tan(δ) losses.

Figure 18 illustrated the broadband spectroscopy of the relative permittivity ($\varepsilon_{r}$) and the dielectric losses (denoted tan(*δ*)) of one-stack devices including a terpolymer or copolymer layer (~9–10 µm thick) printed on using a 125 µm thick PEN substrate. The samples were subjected to different pressures of annealing treatment, i.e., with or without vacuum. Although measurement was carried out in a large frequency range (from 20 Hz to 2 MHz), the mechanical actuation driven from an active guidewire is much lower than 100 Hz. Under such low frequencies, $\varepsilon_{r}$ of the copolymer sample is supposed to be constant, whatever the annealing condition. Regarding the terpolymer, however, a significant increase in permittivity (from 30 to 50 at 20 Hz) was been obtained using the vacuum elaboration method. This phenomenon can be explained by the fact that the vacuum annealing step allowed us to decrease the residue, which resulted in larger chain mobility of the amorphous chain [82,83]. The more mobile the chains are (i.e., the lower the degree of crystallinity), the higher the dielectric constant will be. Consequently, the terpolymer, naturally presenting higher chain mobility than the copolymer, is more greatly impacted by the vacuum annealing treatment. In general, the terpolymer leads to higher permittivity but also more important dielectric losses compared to the copolymer [24].

**Figure 19:**
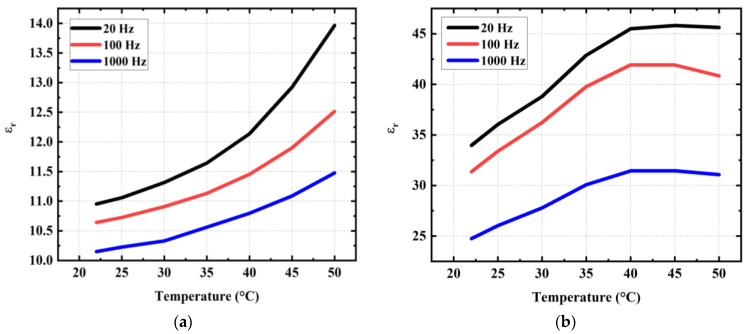
Dielectric permittivity acquired at 20 Hz, 100 Hz, and 1000 Hz of (**a**) PVDF-TrFE copolymer and (**b**) PVDF-TrFE-CTFE terpolymer, annealed at 150 °C without vacuum, and supplied with a 1 V input under temperature variation from 22 to 50 °C.

Given the intended medical application for actuator devices, the influence of temperature on the permittivity was also investigated for both terpolymer and copolymer, annealed without vacuum. As displayed in Figure 19a,b, $\varepsilon_{r}$ of these samples increases with the increasing temperature but not at the same rate. Particularly between room temperature (~23 °C) and average temperature of human bodies (~37 °C), $\varepsilon_{r}$ of the copolymer only augments of one (~8%) while eight (~24%) are augmented in the case of the terpolymer. Above 40 °C, the permittivity of the terpolymer reaches a steady plateau then gradually decreases from 45 °C. Inversely, the copolymer continues rising at a somewhat higher rate (~16%), but its permittivity is still threefold smaller than that of the terpolymer, for a given operating frequency (e.g., 20, 100, and 1000 Hz). On the other hand, the dielectric property of the copolymer is clearly more stable in terms of temperature change and annealing condition (with or without vacuum) with respect to the terpolymer.

**Figure 20:**
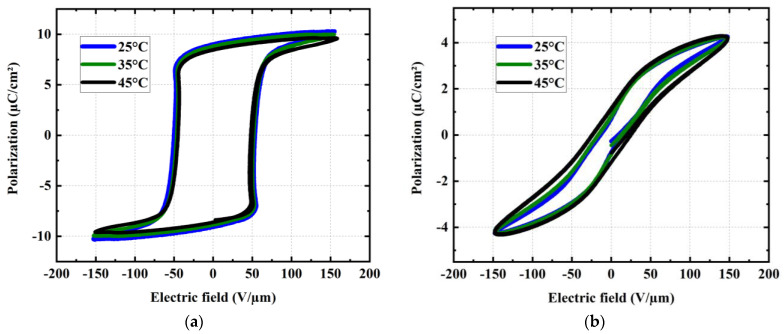
Variation in P-E hysteresis loops of (**a**) PVDF-TrFE copolymer and (**b**) PVDF-TrFE-CTFE terpolymer with temperature set at 25, 35, and 45 °C.

**Figure 21:**
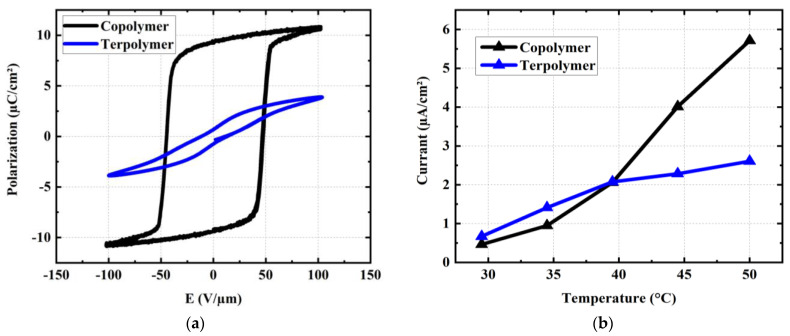
Electrical properties of copolymer and terpolymer samples. (**a**) P-E hysteresis loops measured at 10 Hz. (**b**) Temperature variation in the leakage current density acquired at a DC electric field of 20 V/µm.

In addition, the polarization (µC/cm^2^) as a function of the electric field (E) was investigated, as shown in Figure 20. Measurements were conducted on the same samples used in Figure 19, but under room temperature. A poling procedure that orients the dipoles was performed for all samples (even with the terpolymer, a ferroelectric relaxer) before measuring the P-E hysteresis cycles. Devices were biased at a 10 Hz periodic sinusoidal signal of 20 V/µm amplitude (below the breakdown voltage limit). The voltage was applied for 10 s until the maximum polarization was achieved. Logically, the PVDF-TrFE copolymer exhibits a larger hysteresis area with higher remanent polarization and coercive field as opposed to the PVDF-TrFE-CTFE terpolymer, which retains zero-field polarization. The results shown in Figure 21a allow us to validate the relaxing behavior of the terpolymer versus the ferroelectric characteristics of the copolymer. Moreover, the P-E cycles measured at different temperature of 25 °C, 35 °C, and 45 °C (cf. Figure 20) confirm that the electrical property of both materials is somehow stable within the set temperature range. Interestingly, the terpolymer cycle seems to be slightly larger than the increasing temperature, which might be related to an enhancement in the mobility of the molecular chains.

To verify whether the device could cause trouble for patient in case of permeability defect occurring throughout the guidewire manipulation, the leakage current density (µA/cm^2^) of both polymers was determined. As displayed in Figure 21b, the leakage current, principally caused by the conduction losses of materials, increases with the temperature. This finding can be explained by an increase in the charge mobility with the rise in temperature. Both terpolymer and copolymer generate similar leakage currents under the operating temperature range (i.e., 25–40 °C). Above 40 °C, the conduction losses of the terpolymer rises more quickly as opposed to its counterpart. However, its current level remains negligible (~12.5 µA) compared to the limited threshold that is supposed to be dangerous to patients (~50 µA), according to medical standards (60601-1).

### 3.5. Mechanical Analyses

**Figure 22:**
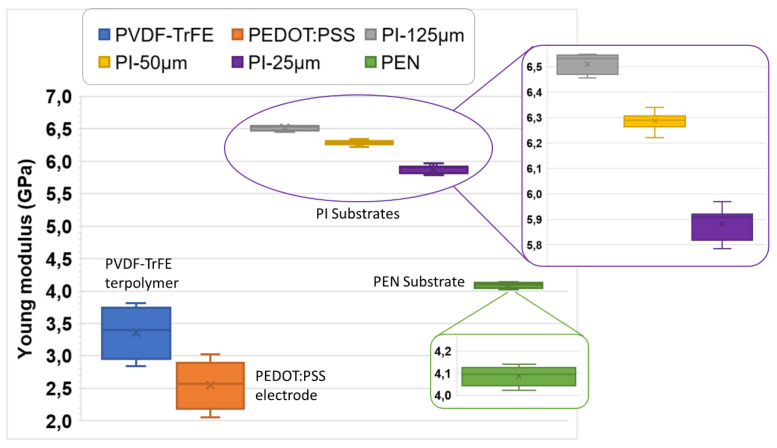
Whisker-box graphs of the Young’s modulus computed through several measurements conducted on different materials including terpolymer, PEDOT electrode, PI, and PEN substrates.

In this study, six materials were used to investigate the impact of mechanical characteristics, which consisted of PVDF-TrFE copolymer, PEDOT:PSS conductor, PEN (125 µm thick), and PI (with different thickness of 25, 50, and 125 µm). For each type of materials, measurements were performed several times at different locations of the sample via the nanoindentation test. The values measured and the average values (used in the various calculations and simulations) are shown in Table S1 of Supplementary Materials. Based on the mechanical setup described in Section 2.3.4, it is possible to induce the Young’s modulus (denoted Y) of the set materials. To better analyze the locality, dispersion, and skewness of the acquired data, a statistical overview using the boxplot graph is illustrated in Figure 22.

The numerical values consisting of the mean value, the standard deviation (*SD*), the maximum, and the minimum values are provided in Table 4. The three quartiles are reported as well, where $Q_{1}$ is the 25th percentile (also called the lower quartile), $Q_{2}$ is the 50th percentile (i.e., the median of the entire dataset), $Q_{3}$ the 75th percentile (also called the upper quartile), and IQR is the interquartile range. To better assess the variability of the data, we provide here an estimation of the quartile coefficient of dispersion (QCD, i.e., given by Equation (12)). The higher the QCD value, the more dispersed the dataset.

(12) $$QCD=\frac{Q_{3}-Q_{1}}{Q_{3}+Q_{1}}=\frac{IQR}{Q_{3}+Q_{1}}$$

The Young’s modulus Y of the PVDF-TrFE-CTFE terpolymer and the PEN (50 µm thick) found in the literature are listed at the two last column of Table 4. As seen, most materials have similar mechanical properties, with Y equal to a few GPa, except the terpolymer which is much more flexible (Y~0.1 GPa). Regarding electroactive polymers, the terpolymer and copolymer cannot be used interchangeably for the same geometry (see Section 3.1). The Poisson’s ratio (ν) of the materials found in the literature is also listed in the last two rows of Table 4. This coefficient is revealed to be constant, regardless of the samples’ thickness.

The detailed data related to the box graph given in Figure 22 and Table 4 leads to the following remarks:

- For all materials, the data distributions are almost symmetrical, as the median (horizontal lines in the whisker box) is close to the mean value (the cross); thus, the skewness should be near to zero.
- All observations did not show any outliers or extremes values (i.e., fall below Q_1_ − 1.5 IQR or above Q_3_ + 1.5 IQR), meaning that the highest and lowest occurring values were within this limit interval.
- Regarding the measures of the PI samples, their thickness somewhat influences the result: the higher the thickness, the higher the Young’s modulus (Y). Although Y is supposed to be an intrinsic property of material (so independent of the sample’s size), in reality, the determination of this parameter is somehow affected by some factors, including the sample’s thickness.
- Concerning the Young’s modulus of the PEN, an important discrepancy (~30%) is observed between the literature and our measurement. This may come from the differences in material and process (grade, homogeneity, dimension, etc.), or differences in technique and condition of measurement.
- PI and PEN samples lead to a very small dispersion of Y with the coefficient QCD < 1%. In the case of the terpolymer and PEDOT:PSS, QCD is revealed to be higher, but still lower than 10%, confirming good repeatability of the data.

### 3.6. Parameters Influencing Tip Deflection

#### 3.6.1. Number of Stacks and Thickness of Substrate/EAP

**Figure 23:**
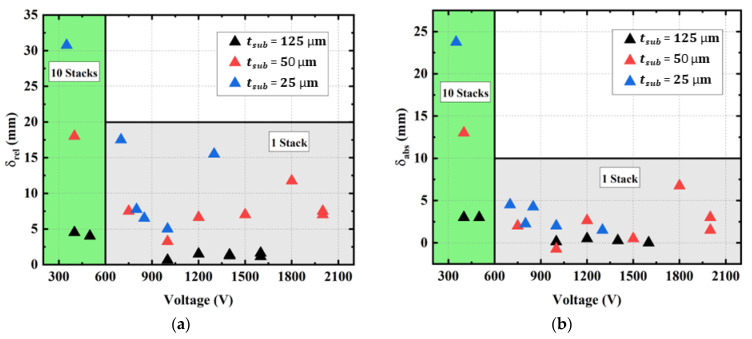
Maximum relative and absolute displacement (respectively, (**a**) and (**b**)) as a function of the applied input voltage for 10-stack copolymer printed on substrate with different thickness of 25, 50, and 125 µm.

Figure 23a depicts the relative displacement (i.e., the difference between the initial and the final position) of the device tip as a function of the input voltage, while Figure 23b shows the absolute displacement (i.e., the final position). Due to manufacturing-induced pre-stress creating a displacement opposite to that generated by the input field, the absolute displacement of the copolymer is somewhat smaller than its relative displacement. As observed, the displacement is enhanced for the devices with thin substrates (e.g., 25 µm), which is contrary to the 125 µm thick substrate that leads to very small deformation, even with a 10-stack pattern. Accordingly, the multi-layered design of the active polymers, when printed on a thin substrate, allowed us to achieve tip deflection under a moderate input voltage. The single-layered (one-stack) design, however, suffering from an ultimately low deformation, needs high-voltage power to achieve the desired deflection. Because of the pre-stressing effect, the final displacement of the one-stack devices (Figure 23b) is still extremely low (≤5 mm), despite thin substrate and high voltage application.

**Figure 24:**
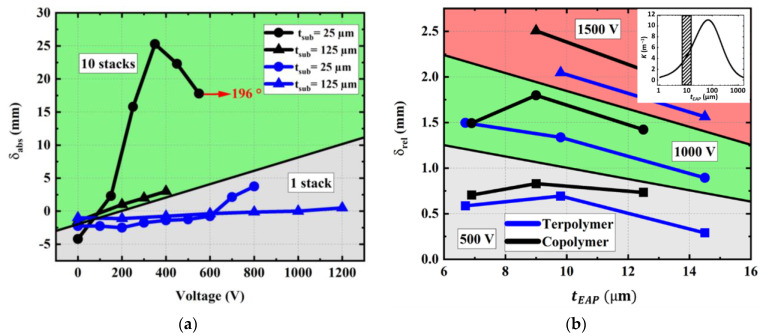
(**a**) Actuation ability under a large range of the input voltage applied to copolymer samples with different stack number (1 or 10) and substrate thickness (25 or 125 µm). (**b**) Relative displacement as a function of the stack thickness at three different input levels (500, 1000, and 1500 V) subjected to 1-stack and 31 mm long devices printed with terpolymer or copolymer deposited on a 125 µm thick PEN substrate.

Figure 24a displays the absolute tip displacement of the one-stack and ten-stack copolymer devices printed on a 25 µm and a 125 µm thick substrate. While Figure 23 gives an overview on the actuation ability of these samples under a large voltage range, Figure 24 takes a closer look within the lower range, for easier visualization and analyses. To some extent, the substrate thickness does not have much influence on the one-stack device, which experiences very small deformation, even with high voltage application. On the other hand, combining thin substrate with several stacks allows us to substantially boost the tip deflection while lowering the input voltage. For the ten stack devices, the maximum displacement is obtained under 400–500 V whereas it is obtained under 800–1200 V in the case of the one-stack devices. Therefore, it is interesting to stack the layers to reduce probability of electrical breakdown of the device while significantly increasing the curvature.

Indeed, when multiple piezoelectric layers are stacked together, they can generate a larger displacement due to the increase in the total electric charge across the thickness of the piezoelectric layers. This phenomenon is known as the “stacking effect” [81]. The stacking effect can be further enhanced by arranging the polarity of the piezoelectric layers in a certain pattern. For example, if the piezoelectric layers are arranged with alternating polarities, the overall displacement of the button can be increased. This is known as the “poling pattern” or “polarization pattern” [81]. However, adding more piezoelectric layers can also increase the stiffness of the guidewire, which may affect the device’s maneuverability. Therefore, it is important to find a balance between the number of layers and mechanical flexibility. In the following simulation results, two stacked piezoelectric layers are enough to achieve the desired displacement.

Figure 24b shows the tip displacement (relative, $\delta_{rel}$) of a one-stack terpolymer and copolymer devices subjected to three different voltage levels including 500, 1000, and 1500 V. The thickness of the stack could be increased by screen-printing several electroactive layers via the same mask. There is no clear trend of $\delta_{rel}$ as a function of the EAP thickness (denoted $t_{EAP}$, 6 µm to 15 µm). However, it can be revealed that the empirical displacement is below the optimum value predicted from the analytical and numerical models previously described in Section 3.1. The pre-stressing of the samples is one of the reasons for this inconsistency. Moreover, homogeneous materials together with other assumptions (e.g., permittivity, polarization, and leakage are independent of $t_{EAP}$) were used to model the device, which is somewhat different to the reality. A solution to improve the reliability of the finite element (FE) and analytical models would be to incorporate the heterogeneity within the polymer layers (e.g., discretizing these layers into sub-layers with different properties). Due to the random nature of screen-printing additive manufacturing (automatically performed at the lab scale), it is difficult to guarantee a constant quality among layers.

It has been shown in Section 3.1 that there is an optimum value of $t_{EAP}$ with which the tip displacement is maximum. To a certain extent, the optimum point does depend on the substrate thickness, so the thicker substrate should be combined with the thicker EAP layer to achieve enhanced actuation performance. It is highlighted that, under the same configuration of the input voltage and sample thickness, the copolymer leads to better piezoelectric response with respect to the terpolymer. The following analysis allows us to better address this issue.

#### 3.6.2. Nature of Substrate and EAP

**Figure 25:**
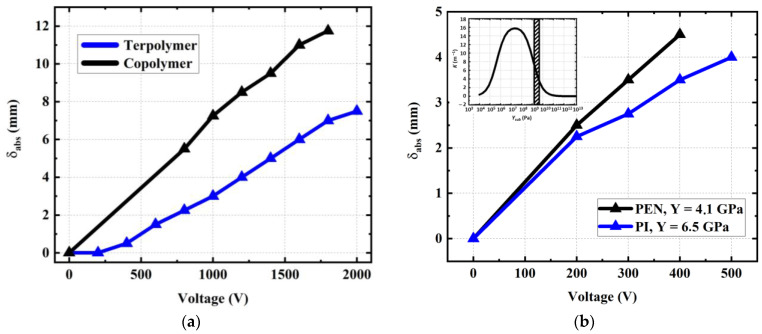
(**a**) Influence of EAP material on the absolute tip displacement of 1-stack devices printed on substrates of 50 µm thick. (**b**) Influence of the substrate nature on the absolute tip displacement of 10-stack copolymer devices printed on 125 µm thick substrates made of PEN and PI.

Figure 25a illustrates the relative tip deflection ($\delta_{rel}$) of a one-stack terpolymer and copolymer (with 12–14 µm thick) printed on 50 µm PEN substrates. Similar to the analytical models (see Figure 10), those designed with thin substrates lead to a better electromechanical response. It is worth noting that the copolymer devices induce a higher tip displacement, regardless of what substrate thickness and input voltage are chosen. Particularly, in the case of a thin substrate, the displacement is almost double for the copolymer as opposed to the terpolymer.

Figure 25b shows the influence of the substrate nature (PI or PEN) on the actuation ability, where the copolymer/PEN device leads to a better displacement with respect to the PI counterpart and, therefore, better curvature. Indeed, the PEN, with its smaller stiffness (4.1 GPa versus 6.5 GPa, as in the case of the PI), facilitates the bending of the structure. However, in this configuration (10 stacks printed on a 125 µm substrate), the substrate should be somewhat stiffer than the EAP layer (see Table 4) in such a way that it is sufficiently resistant to correctly support device bending. Otherwise, the energy generated by the actuator only lengthens the device instead of making it bend.

#### 3.6.3. Device Slenderness

**Figure 26:**
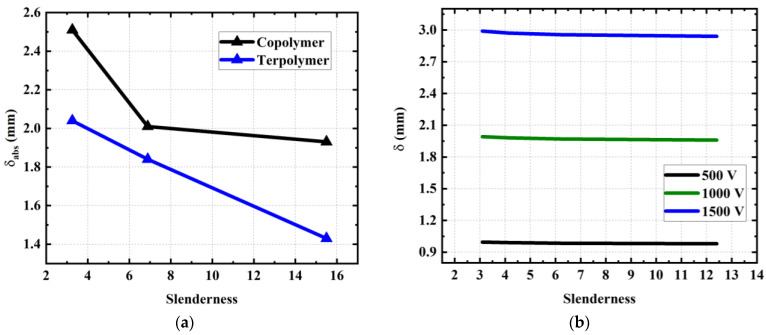
Effect of the geometrical parameters on the actuation performance: (**a**) tip displacement of terpolymer and copolymer actuators subjected to 1500 V as a function of the slenderness. (**b**) tip displacement of copolymer device versus its width according to FEM simulation.

This study aims to highlight the effect of the device’s slenderness (i.e., defined as the ratio between length and width) on the actuator behavior. The experimental result illustrated in Figure 26a indicates that the absolute tip displacement ($\delta_{abs\;}$) decreases with slender architecture, which is not in the case in the 2D analytic model. The fact is that a real device is surrounded by a substrate 1 mm wide (to avoid electrical short-circuits), which, in turn, affects the slenderness as well as the active-material area with respect to the total surface of the device. Consistent with the previous observations, the deflection generated by the copolymer device is superior to the one of the terpolymer, regardless of the slenderness. To dissociate the effect of slenderness from that of the surrounding substrate, a 3D simulation model was conducted via COMSOL software under different input voltage amplitudes. As suggested in Figure 26b, the tip displacement computed from the numerical method is almost constant as a function of the width (i.e., inversely proportional to the slenderness). More precisely, a decrease of only 2% in $\delta_{abs\;}$ has been recorded, whereas it was 40% in the experiment. Hence, this discrepancy is supposed to be mainly manifested by the surrounding substrate (95% contribution) rather than the geometric slenderness (5% contribution). Also, the small influence of the slenderness found through the FEM allows us to validate the relevance of the developed analytical model. It is worth noting that for arterial navigation slimmer devices exhibit doubly advantageous. In addition to being able to reach finer blood vessels (and, thus, broaden the guide’s possibilities), these devices reduce the EAP surface, leading to lower leakage currents and decreasing the risk to patients.

### 3.7. Design Guideline of a Smart Guidewire-Based Printed EAP

From a manufacturing point of view, it is not possible to compare the copolymer PVDF-TrFE and the terpolymer PVDF-TrFE-CTFE at all equal parameters. Although the printing process of the EAP inks are conducted in identical conditions, the terpolymer film, with its higher viscosity, tends to be thicker (10–20%) than the copolymer one. Whatever the thickness of the EAP or the substrate (Section 3.6.1), or even the sample slenderness (Section 3.6.3), the copolymer is revealed to be a higher-performance actuator.

From a geometrical point of view, the previous analyses (Section 3.1 and Section 3.6) have pointed out that, for a given substrate thickness, there exists an optimum electroactive thickness or an optimum number of stack within which the device can reach the maximum tip displacement. Using a multi-stack pattern instead of a one-stack pattern would considerably lower the input voltage, thus decrease the breakdown field of the EAP, while obtaining better curvature for the guidewire. Such achievements are extremely interesting for the medical field as high voltage sources near to the patient environment is, to some extent, constraining and sometimes not allowed. The slenderness of the device has been shown to slightly impact the actuation behavior if the electroactive layer is printed as close as possible to the surrounding substrate.

From a material point of view, it would be interesting to decrease the stiffness of the substrate and/or increase the stiffness of the electroactive polymer to further enhance the curvature as well as correctly bend the device. It would also be ensured that the piezoelectric and the electrostrictive coefficient, respectively, denoted as d_31_ and M_31_, are not drastically altered. The copolymer, which is ultimately much stiffer than the terpolymer, seems to be an appropriate choice. Regarding the substrates used in this study, the polyethylene-naphthalene (PEN), thanks to its lower Young’s modulus, is probably preferred more than the polyimide (PI). In any case, the substrate, playing a role of support, might be somewhat more rigid than the electroactive layer so that the energy is efficiently transferred to bend the structure. Practical tests revealed that the copolymer/PEN device leads to slightly superior displacement than the copolymer/PI counterpart.

From a medical point of view, the PI material has been demonstrated to be medical grade and, thus, is largely used on an industrial scale. Therefore, the PI is selected for the development of our target device. Both copolymer and terpolymer were shown to be biocompatible and sterilizable [89,90,91], but the copolymer was finally chosen due to its higher ability to bend.

To conclude, a guideline used for the design strategy of a smart guidewire, or other bending devices, can be proposed according to the above analyses: First, the nature of the substrate and its thickness are selected since the thickness and mechanical properties are imposed by the substrate manufacturer. The choice of the substrate parameters must be set in accordance with the targeted bending angle: a larger angle implies the use of a smaller substrate thickness. Second, the nature of the EAP layer is chosen, which ultimately depends on the Young’s modulus of the substrate. Third, the selection of the electrode ink, mainly based on its viscosity, adhesion, and electrical conductivity, is also considered. In the case of the sensor and/or actuator network (i.e., out of scope of this study), where the electrode pattern is somehow complex, the design rules together with the influence of the electrodes on the device’s response must be thoroughly investigated. Last but not least, deep analyses on the bending performance are carried out, which are strongly impacted by several material’s parameters (e.g., geometrical, mechanical, and electrical characteristics) as well as the design architecture (e.g., multi-stack).

**Figure 27:**
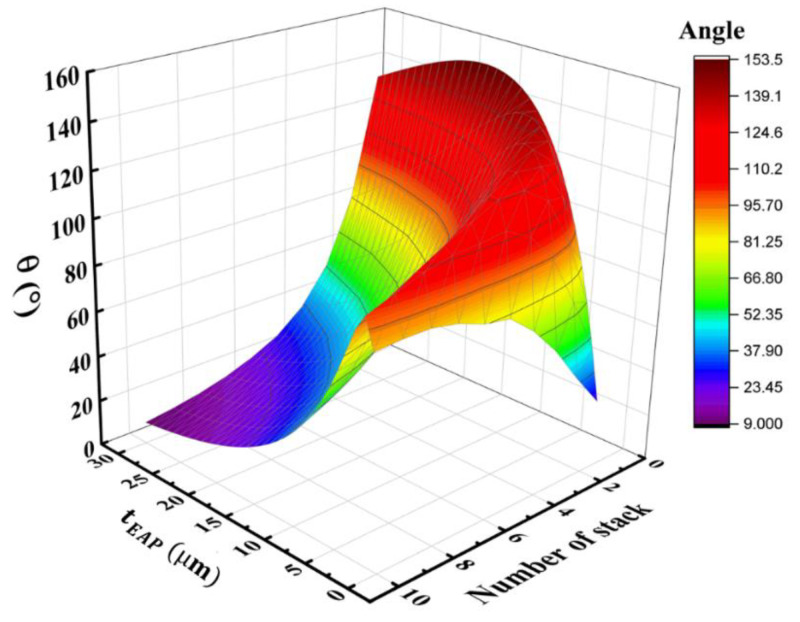
Bending angle predicted from the analytical model as a function of the EAP thickness and the number of stacks. The theoretical model, consisting of single-stack or multi-stack copolymer deposited on a 25 µm PI substrate, is subjected to a constant electric field of 200 V/µm.

For instance, the last issue can be partially explored using analytical models or the finite element method (FEM) to estimate the bending angle (denoted θ) as a function of the EAP thickness of each layer ($t_{EAP}$ and the number of the layers (N). The bending angle $\theta =f(t_{EAP},N)$ shown in Figure 27 allows us to confirm that both $t_{EAP}\;$and N parameters have an ultimate influence on the bending angle θ. As the model depends on many other parameters, it is necessary to understand their impact on the bending response to optimize the design as well as to improve the target performance. By selecting adequate parameters, it is possible to achieve a large angle range and/or displacement of the device. Overall, Figure 27 reveals that, for a given target angle, there are numerous ways of sizing the actuator. Indeed, to achieve the maximum angle (and the curvature as well), it is possible to use either several thin stacks or a few thick stacks. The difference involves the voltage needed to actuate the device in such a way that the thicker the stack, the greater the voltage. For an easier manufacturing process and simpler design, the thick stacks are revealed to be an appropriate solution with a few number of electrodes. Nonetheless, for medical instrumentation, where the safety of patients is one of the main concerns, thin stacks are preferable to limit the voltage to as low as possible.

## 4. Conclusions

This study reported on the development of a novel smart steerable-guidewire-based EAP that was demonstrated to be a relevant candidates for cardiovascular Micro-Invasive Surgery (MIS). For the sake of simplicity, experimental characterizations were carried out on a simple cantilever–plane structure (instead of the tubular-shaped wire), which leads to the following relevant remarks:

- Structural analyses via XRD confirmed that the annealing treatment under vacuum and high temperature (150 °C) resulted in the best homogeneity and crystallization for the polymers layers.
- Observation-based SEM of the cross section of the printed sample (including an EAP layer sandwiched between the substrate and the electrodes) allowed us to valid the good print quality of each layer.
- AFM images highlighted the small roughness with very few defects of the printed polymer surface, confirming the possibility of performing the staking multi-layer design.
- Broadband dielectric measurement pointed out that, compared to the copolymer, the terpolymer leads to higher permittivity but less stability vis a vis the temperature change and the annealing pressure.
- Polarization hysteresis cycles allowed us to confirm the relaxing behavior of the terpolymer and the ferroelectric characteristics of the copolymer. The electrical properties of both materials were revealed to be stable at a temperature range from 25 °C to 45 °C.
- Mechanical characterization indicated that the Young’s modulus of the copolymer and the substrates are of the same order, which is necessary to achieve good bending behavior.

To optimize the curvature of the devices and quantify the influence of the parameters relating to the material’s properties and geometrics, an analytical model as well as a finite element model (FEM) were developed; both have been shown to be reliable when compared to practical tests. It has been demonstrated that the copolymer leads to a better displacement response with respect to the terpolymer. Experimental results revealed a large enhancement of the tip deflection under a relatively low electric field for the multi-layered design of the copolymer, when printed on a thin substrate. These results confirm the high potential of the developed material for real-world actuator applications, especially in multifunctional flexible electroactive devices. Regarding an important number of parameters as well as specifications imposed by the medical standards, a design guideline was proposed to better understand the influence of those parameters on the actuation of the device, making it possible to simplify the optimization analyses while respecting medical constrains.

Although the analytical and simulation models remain reliable, some results showed the limits of the assumptions made, particularly related to the homogeneity of the materials. These models can be improved by integrating, for instance, gradients of certain parameters such as permittivity and Young’s modulus. This research initiates the first key step in designing an electrical steerable guidewire accompanied by a comprehensible and methodologic framework. In advancing towards the development of a real prototype as an early step in the industrialization of this research, there are still challenges to overcome, notably the repeatability of the bending in voltage, the sealing, and the force developed by the device against the blood flow. In the future, the manual tools currently used by surgeons will be replaced by soft, electrically controllable devices that will offer new perspectives to cardiovascular surgery.

## Figures and Tables

**Table 1:** Characteristics of print masks.

Mask	Mesh	Φ Thread [µm]	Material	Thickness [µm]
A	235	24	Stainless steel	1
B	230	36	Stainless steel	2.5
C	43	80	Polyester	4.4

**Table 2:** Material parameters used in the COMSOL simulation.

Material	Value	Title 3
PEN substrate		
Poisson’s Raito	0.4	-
Young’s modulus	4.1	GPa
PI substrate		
Poisson’s Raito	0.4	-
Young’s modulus	6.5	GPa
PVDF-TrFE copolymer		
Density	1770	kg.m^−3^
Poisson’s Raito	0.4	-
Young’s modulus	2.4	GPa
Length (L)	31	Mm
Relative permittivity *ε_r_*	12	-
Piezoelectric coefficient *d*_31_	15	pC/N
Piezoelectric coefficient *d*_32_	2	pC/N
Piezoelectric coefficient *d*_33_	-28	pC/N
PEDOT:PSS electrode		
Density	1180	-
Poisson’s Raito	0.35	-
Young’s modulus	2.5	GPa
Thickness	1	µm

**Table 3:** Size of crystallized domains according to methods of elaboration.

Pressure	Temperature	Domain Size (nm)
Ambient	150 °C	8
Ambient	70 °C	10.5
Vacuum	150 °C	12
Vacuum	Ambient	7.5

**Table 4:** Mechanical parameters of materials developed throughout this study and those collected from the literature (given the corresponding reference).

		PVDF-TrFE	PEDOT:PSS	PI-125 µm	PI-50 µm	PI-25 µm	PEN-125 µm	PEN-50 µm	PVDF-TrFE-CTFE
Young’s Modulus	Mean (GPa)	3.308	2.547	6.510	6.286	5.882	4.087	5.3 [84]	0.103 [85]
	SD (GPa)	0.289	0.287	0.036	0.026	0.054	0.035	-	-
	Min (GPa)	2.839	2.050	6.455	6.222	5.785	4.023	-	-
	Max (GPa)	3.815	3.026	6.548	6.340	5.969	4.141	-	-
	Q_1_ (GPa)	3.079	2.326	6.473	6.272	5.836	4.065	-	-
	Q_2_ (GPa)	3.282	2.566	6.532	6.290	5.908	4.096	-	-
	Q_3_ (GPa)	3.522	2.766	6.543	6.304	5.921	4.111	-	-
	IQR (GPa)	0.443	0.440	0.069	0.032	0.085	0.046	-	-
	QCD (%)	6.71	8.64	0.53	0.25	0.72	0.56	-	-
Poisson coefficient	ν	0.28 [86]	0.33 [87]	0.34 [88]			0.33 [84]		0.48 [74]

## Data Availability

Data are contained within the article.

## Supplementary Materials

The following supporting information can be downloaded at: https://www.mdpi.com/article/10.3390/ma17092135/s1, S1: Results of PEDOT:PSS, PVDF-TrFE, PI and PEN nano-indentation measurement; Table S1: Nano-identation measurement results; S2: Analytical model development.
