# Supplementary material for: Design Optimization of Printed Multi-Layered Electroactive Actuators Used for Steerable Guidewire in Micro-Invasive Surgery
Source: Materials (Basel). 2024 May 2;17(9):2135. doi: 10.3390/ma17092135 (PMC11085776; doi:10.3390/ma17092135)
Supplement: Supplementary file 1 [file materials-17-02135-s001.zip › materials-2952837-supplementary.pdf]

<u>Supporting information</u>

S1 : Results of PEDOT:PSS, PVDF-TrFE, PI and PEN nano-indentation measurement

Young's modulus values were measured several times at different locations on the sample. The values measured and the average values (used in the various calculations and simulations) are shown in Table S1.

Measurement	$Y_{PVDF\text{-}TrFE}$	$Y_{PEDOT:PSS}$	$Y_{PI125\mu m}$	$Y_{PI50\mu m}$	$Y_{PI25\mu m}$	Y_{PEN}
**1**	3.8153	2.56604	6.54554	6.28066	5.92086	4.09562
**2**	3.28228	2.04968	6.45485	6.33955	5.96882	4.14131
**3**	2.83921	2.32618	6.46876	6.30104	5.81924	4.02294
**4**	3.52214	3.02577	6.54008	6.26402	5.78495	4.06516
**5**	3.07907	2.76592	6.53188	6.30675	5.92086	4.11085
**6**	-	-	6.47821	6.22153	5.85228	-
**7**	-	-	6.54828	6.29011	5.90844	-
**Average value**	3.3	2.5	6.5	6.3	5.9	4.1

Table S1 : Nano-identation measurement results

S2 : Analytical model development

The thickness of the substrate is t_{sub}, the *(N+1)* electrodes have the same thickness, t_{elec} and t_{diel} denotes the thickness of the *N* dielectric layers.

In addition to previous assumption, we assume that the device has a longitudinal (along x-axis) strain and a flexural strain. Moreover, we assume that the multilayer bends in an arc of circle [34], [17]. Finally we assume that the neutral axis is the boundary between the substrate and the first electrode [17].

Under these assumptions, the stress in the different layers can be written as:

$$\begin{cases} T_{sub} = Y_{sub}(Kz + S_0) \\ T_{elec,k} = Y_{elec}(Kz + S_0) \\ T_{EAP,k} = Y_{EAP}(Kz + S_0 - S_d) \end{cases}$$

With:

$$\begin{cases} Y_i \text{ the Young's modulus } [Pa] \\ K \text{ the curvature } [m^{-1}] \\ S_0 \text{ longitudinal strain (along } x-\text{axis) } [-] \\ S_d \text{ the dielectric's strain (generated by piezoelectricity or electrostriction) } [-] \\ z_{a,b} \text{ the position along } z-\text{axis}, b \text{ is the layer number} \end{cases}$$

For an electrostrictive PVDF-TRFE-CTFE material (without temperature change), strain and electric displacement are expressed as [18]:

$$\begin{cases} S_{31} = M_{31}E^2 + sT_{31} \\ D_{31} = \varepsilon_0\varepsilon_r E + 2M_{31}ET_{31} \end{cases}$$

With:

$$\begin{cases} M_{31} \text{the electrostricitive coefficient} \\ S_{31} \text{ the strain in direction 1 due to a solicitation in direction 3} \\ D_{31} \text{ the electric displacement in direction 1 due to a solicitation in direction 3} \\ s \text{ the mechanical compliance (from compliance tensor)} \\ E \text{ the electric field} \\ T_{31} \text{the stress in direction 1 due to a solicitation in direction 3} \\ \varepsilon_0\varepsilon_r \text{ the permittivity of the dielectric} \end{cases}$$

In absence of external stress, the strain can be written as:

$$S_{31} = M_{31}E^2$$

For a piezoelectric PVDF-TRFE material (without temperature change), strain and electric displacement are expressed as [20]:

$$\begin{cases} S_{31} = d_{31}E + sT_{31} \\ D_{31} = \varepsilon_0\varepsilon_r E + d_{31}T_{31} \end{cases} \text{ with } d_{31} \text{the piezoelectric coefficient}$$

In absence of external stress, the strain can be written as:

$$S_{31} = d_{31}E$$

Forces equilibrium:

$$\int_{-t_{sub}}^{0} T_{sub}.dz + \sum_{k=1}^{N+1} \int_{z_{EAP,(k-1)}}^{z_{elec,k}} T_{elec,k}.dz + \sum_{k=1}^{N} \int_{z_{elec,k}}^{z_{EAP,k}} T_{EAP,k}.dz = 0$$

$$\leftrightarrow \int_{-t_{sub}}^{0} Y_{sub}(Kz + S_0).dz + \sum_{k=1}^{N+1} \int_{z_{EAP,(k-1)}}^{z_{elec,k}} Y_{elec}(Kz + S_0).dz + \sum_{k=1}^{N} \int_{z_{elec,k}}^{z_{EAP,k}} Y_{EAP}(Kz + S_0 - S_d).dz = 0$$

On the assumption that the stiffness is constant in a layer:

$$\leftrightarrow Y_{sub} \int_{-t_{sub}}^{0} Kz.dz + Y_{sub} \int_{-t_{sub}}^{0} S_0.dz + \sum_{k=1}^{N+1} Y_{elec} \int_{z_{EAP,(k-1)}}^{z_{elec,k}} Kz.dz + \sum_{k=1}^{N+1} Y_{elec} \int_{z_{EAP,(k-1)}}^{z_{elec,k}} S_0.dz + \sum_{k=1}^{N} Y_{EAP} \int_{z_{elec,k}}^{z_{EAP,k}} Kz.dz + \sum_{k=1}^{N} Y_{EAP} \int_{z_{elec,k}}^{z_{EAP,k}} S_0.dz = \sum_{k=1}^{N} Y_{EAP} \int_{z_{elec,k}}^{z_{EAP,k}} S_d.dz$$

$$\leftrightarrow Y_{sub} \int_{-t_{sub}}^{0} Kz.dz + Y_{sub}S_0t_{sub} + \sum_{k=1}^{N+1} Y_{elec} \int_{z_{EAP,(k-1)}}^{z_{elec,k}} Kz.dz + (N+1)t_{elec}Y_{elec}S_0 + \sum_{k=1}^{N} Y_{EAP} \int_{z_{elec,k}}^{z_{EAP,k}} Kz.dz + Nt_{EAP}E_{EAP}S_0 = \sum_{k=1}^{N} Y_{EAP} \int_{z_{elec,k}}^{z_{EAP,k}} S_d.dz$$

On the assumption that the electric filed is the same in every layer:

$$\leftrightarrow KY_{sub}\left[\frac{z^2}{2}\right]_{-t_{sub}}^{0} + Y_{sub}S_0t_{sub} + KY_{elec}\sum_{k=1}^{N+1}\left[\frac{z^2}{2}\right]_{z_{EAP,(k-1)}}^{z_{elec,k}} + (N+1)t_{elec}Y_{elec}S_0 + KY_{EAP}\sum_{k=1}^{N}\left[\frac{z^2}{2}\right]_{z_{elec,k}}^{z_{EAP,k}} + Nt_{EAP}Y_{EAP}S_0 = Nt_{EAP}Y_{EAP}S_d$$

$$\leftrightarrow S_0(Y_{sub}t_{sub} + (N+1)t_{elec}Y_{elec} + Nt_{EAP}Y_{EAP}) + K\left(Y_{sub}\left[\frac{z^2}{2}\right]_{-t_{sub}}^{0} + Y_{elec}\sum_{k=1}^{N+1}\left[\frac{z^2}{2}\right]_{z_{EAP,(k-1)}}^{z_{elec,k}} + Y_{EAP}\sum_{k=1}^{N}\left[\frac{z^2}{2}\right]_{z_{elec,k}}^{z_{EAP,k}}\right) = Nt_{EAP}Y_{EAP}S_d$$

$$\leftrightarrow AS_0 + BK = C$$

$$with \begin{cases} A = (E_{sub}t_{sub} + (N+1)t_{elec}Y_{elec} + Nt_{EAP}Y_{EAP}) \\ B = \left(Y_{sub}\left[\frac{z^2}{2}\right]_{-t_{sub}}^{0} + Y_{elec}\sum_{k=1}^{N+1}\left[\frac{z^2}{2}\right]_{z_{EAP,(k-1)}}^{z_{elec,k}} + Y_{EAP}\sum_{k=1}^{N}\left[\frac{z^2}{2}\right]_{z_{elec,k}}^{z_{EAP,k}}\right) \\ C = Nt_{EAP}Y_{EAP}S_d \end{cases}$$

<u>Moments equilibrium:</u>

$$\int_{-t_{sub}}^{0} T_{sub}.z.dz + \sum_{k=1}^{N+1}\int_{z_{z_{EAP,(k-1)}}}^{z_{elec,k}} T_{elec,k}.z.dz + \sum_{k=1}^{N}\int_{z_{elec,k}}^{z_{EAP,k}} T_{EAP,k}.z.dz = 0$$

$$\leftrightarrow \int_{-t_{sub}}^{0} Y_{sub}(Kz + S_0).z.dz + \sum_{k=1}^{N+1}\int_{z_{EAP,(k-1)}}^{z_{elec,k}} Y_{elec}(Kz + S_0).z.dz + \sum_{k=1}^{N}\int_{z_{elec,k}}^{z_{EAP,k}} Y_{EAP}(Kz + S_0 - S_d).z.dz = 0$$

$$\leftrightarrow \int_{-t_{sub}}^{0} Y_{sub}Kz^2.dz + \int_{-t_{sub}}^{0} Y_{sub}S_0 z.dz + \sum_{k=1}^{N+1}\int_{z_{EAP,(k-1)}}^{z_{elec,k}} Y_{elec}Kz^2.dz + \sum_{k=1}^{N+1}\int_{z_{EAP,(k-1)}}^{z_{elec,k}} Y_{elec}S_0 z.dz + \sum_{k=1}^{N}\int_{z_{elec,k}}^{z_{EAP,k}} Y_{EAP}Kz^2.dz + \sum_{k=1}^{N}\int_{z_{elec,k}}^{z_{EAP,k}} Y_{EAP}S_0 z.dz = \sum_{k=1}^{N}\int_{z_{elec,k}}^{z_{EAP,k}} Y_{EAP}S_d z.dz$$

$$\leftrightarrow Y_{sub}K\frac{{t_{sub}}^3}{3} - Y_{sub}S_0\frac{{t_{sub}}^2}{2} + Y_{elec}K\sum_{k=1}^{N+1}\left[\frac{z^3}{3}\right]_{z_{EAP,(k-1)}}^{z_{elec,k}} + Y_{elec}S_0\sum_{k=1}^{N+1}\left[\frac{z^2}{2}\right]_{z_{EAP,(k-1)}}^{z_{elec,k}} + Y_{EAP}K\sum_{k=1}^{N}\left[\frac{z^3}{3}\right]_{z_{elec,k}}^{z_{EAP,k}} + Y_{EAP}S_0\sum_{k=1}^{N}\left[\frac{z^2}{2}\right]_{z_{elec,k}}^{z_{EAP,k}} = Y_{EAP}S_d\sum_{k=1}^{N}\left[\frac{z^2}{2}\right]_{z_{elec,k}}^{z_{EAP,k}}$$

$$\leftrightarrow S_0\left(Y_{elec}\sum_{k=1}^{N+1}\left[\frac{z^2}{2}\right]_{z_{EAP,(k-1)}}^{z_{elec,k}}+Y_{EAP}\sum_{k=1}^{N}\left[\frac{z^2}{2}\right]_{z_{elec,k}}^{z_{EAP,k}}-Y_{sub}\frac{{t_{sub}}^2}{2}\right)$$
$$+K\left(Y_{sub}\frac{{t_{sub}}^3}{3}+Y_{elec}\sum_{k=1}^{N+1}\left[\frac{z^3}{3}\right]_{z_{EAP,(k-1)}}^{z_{elec,k}}+Y_{EAP}\sum_{k=1}^{N}\left[\frac{z^3}{3}\right]_{z_{elec,k}}^{z_{EAP,k}}\right)$$
$$=Y_{EAP}S_d\sum_{k=1}^{N}\left[\frac{z^2}{2}\right]_{z_{elec,k}}^{z_{EAP,k}}$$

$$\leftrightarrow DS_0+FK=G\ with\ \begin{cases} D=\left(Y_{elec}\sum_{k=1}^{N+1}\left[\frac{z^2}{2}\right]_{z_{EAP,(k-1)}}^{z_{elec,k}}+Y_{EAP}\sum_{k=1}^{N}\left[\frac{z^2}{2}\right]_{z_{elec,k}}^{z_{EAP,k}}-Y_{sub}\frac{{t_{sub}}^2}{2}\right)\\ F=\left(Y_{sub}\frac{{t_{sub}}^3}{3}+Y_{elec}\sum_{k=1}^{N+1}\left[\frac{z^3}{3}\right]_{z_{EAP,(k-1)}}^{z_{elec,k}}+Y_{EAP}\sum_{k=1}^{N}\left[\frac{z^3}{3}\right]_{z_{elec,k}}^{z_{EAP,k}}\right)\\ G=Y_{EAP}S_d\sum_{k=1}^{N}\left[\frac{z^2}{2}\right]_{z_{elec,k}}^{z_{EAP,k}}\end{cases}$$

Combining equations of moments and forces equilibrium:

$$\begin{cases}AS_0+BK=C\\ DS_0+FK=G\end{cases}$$

$$\leftrightarrow\begin{cases}K=\frac{CD-AG}{BD-AF}\\ S_0=\frac{C-B\left(\frac{CD-AG}{BD-AF}\right)}{A}\end{cases}$$

$$
\leftrightarrow \begin{cases}
K = \dfrac{Nt_{EAP}Y_{EAP}S_d\left(Y_{elec}\sum_{k=1}^{N+1}\left[\frac{z^2}{2}\right]_{z_{EAP,(k-1)}}^{z_{elec,k}} + Y_{EAP}\sum_{k=1}^{N}\left[\frac{z^2}{2}\right]_{z_{elec,k}}^{z_{EAP,k}} - Y_{sub}\frac{{t_{sub}}^2}{2}\right) - (E_{sub}t_{sub} + (N+1)t_{elec}Y_{elec} + Nt_{EAP}Y_{EAP})Y_{EAP}S_d\sum_{k=1}^{N}\left[\frac{z^2}{2}\right]_{z_{elec,k}}^{z_{EAP,k}}}{\left(Y_{sub}\left[\frac{z^2}{2}\right]_{-t_{sub}}^{0} + Y_{elec}\sum_{k=1}^{N+1}\left[\frac{z^2}{2}\right]_{z_{EAP,(k-1)}}^{z_{elec,k}} + Y_{EAP}\sum_{k=1}^{N}\left[\frac{z^2}{2}\right]_{z_{elec,k}}^{z_{EAP,k}}\right)\left(Y_{elec}\sum_{k=1}^{N+1}\left[\frac{z^2}{2}\right]_{z_{EAP,(k-1)}}^{z_{elec,k}} + Y_{EAP}\sum_{k=1}^{N}\left[\frac{z^2}{2}\right]_{z_{elec,k}}^{z_{EAP,k}} - Y_{sub}\frac{{t_{sub}}^2}{2}\right) - (E_{sub}t_{sub} + (N+1)t_{elec}Y_{elec} + Nt_{EAP}Y_{EAP})\left(Y_{sub}\frac{{t_{sub}}^3}{3} + Y_{elec}\sum_{k=1}^{N+1}\left[\frac{z^3}{3}\right]_{z_{EAP,(k-1)}}^{z_{elec,k}} + Y_{EAP}\sum_{k=1}^{N}\left[\frac{z^3}{3}\right]_{z_{elec,k}}^{z_{EAP,k}}\right)} \\
\varepsilon_0 = \dfrac{Nt_{EAP}Y_{EAP}S_d - \left(Y_{sub}\left[\frac{z^2}{2}\right]_{-t_{sub}}^{0} + Y_{elec}\sum_{k=1}^{N+1}\left[\frac{z^2}{2}\right]_{z_{EAP,(k-1)}}^{z_{elec,k}} + Y_{EAP}\sum_{k=1}^{N}\left[\frac{z^2}{2}\right]_{z_{elec,k}}^{z_{EAP,k}}\right)\left(\dfrac{Nt_{EAP}Y_{EAP}S_d\left(Y_{elec}\sum_{k=1}^{N+1}\left[\frac{z^2}{2}\right]_{z_{EAP,(k-1)}}^{z_{elec,k}} + Y_{EAP}\sum_{k=1}^{N}\left[\frac{z^2}{2}\right]_{z_{elec,k}}^{z_{EAP,k}} - Y_{sub}\frac{{t_{sub}}^2}{2}\right) - (E_{sub}t_{sub} + (N+1)t_{elec}Y_{elec} + Nt_{EAP}Y_{EAP})Y_{EAP}S_d\sum_{k=1}^{N}\left[\frac{z^2}{2}\right]_{z_{elec,k}}^{z_{EAP,k}}}{\left(Y_{sub}\left[\frac{z^2}{2}\right]_{-t_{sub}}^{0} + Y_{elec}\sum_{k=1}^{N+1}\left[\frac{z^2}{2}\right]_{z_{EAP,(k-1)}}^{z_{elec,k}} + Y_{EAP}\sum_{k=1}^{N}\left[\frac{z^2}{2}\right]_{z_{elec,k}}^{z_{EAP,k}}\right)\left(Y_{elec}\sum_{k=1}^{N+1}\left[\frac{z^2}{2}\right]_{z_{EAP,(k-1)}}^{z_{elec,k}} + Y_{EAP}\sum_{k=1}^{N}\left[\frac{z^2}{2}\right]_{z_{elec,k}}^{z_{EAP,k}} - Y_{sub}\frac{{t_{sub}}^2}{2}\right) - (E_{sub}t_{sub} + (N+1)t_{elec}Y_{elec} + Nt_{EAP}Y_{EAP})\left(Y_{sub}\frac{{t_{sub}}^3}{3} + Y_{elec}\sum_{k=1}^{N+1}\left[\frac{z^3}{3}\right]_{z_{EAP,(k-1)}}^{z_{elec,k}} + Y_{EAP}\sum_{k=1}^{N}\left[\frac{z^3}{3}\right]_{z_{elec,k}}^{z_{EAP,k}}\right)}\right)}{(E_{sub}t_{sub} + (N+1)t_{elec}Y_{elec} + Nt_{EAP}Y_{EAP})}
\end{cases}
$$
